# Isolation, identification, and whole-genome sequencing of *Streptomyces rochei* FE-3-1 against *Pyricularia oryzae*

**DOI:** 10.1371/journal.pone.0331386

**Published:** 2025-10-29

**Authors:** Dongxia Du, Zhuo Yi, Shiping Shan, Shuaishuai Gao, Mengyuan Yu, Bin Wang

**Affiliations:** 1 Hunan Institute of Microbiology, Changsha, Hunan, China; 2 Hunan Engineering and Technology Research Center of Agricultural Microbiology Application, Changsha, Hunan, China; 3 Yiyang Open University, Yiyang, Hunan, China; Southern Federal University Academy of Biology and Biotechnology named after D I Ivanovsky: Uznyj federal'nyj universitet Akademia biologii i biotehnologii im D I Ivanovskogo, RUSSIAN FEDERATION

## Abstract

*Streptomyces* are significant producers of antimicrobial secondary metabolites. In this study, a *Streptomyces rochei* FE-3–1was isolated from the rhizosphere of rice plants, and its metabolites exhibited potent antagonistic activity against plant pathogen *Pyricularia oryzae*. However, the genome sequence of the strain has not been reported to date. Whole genome sequencing and genome mining were conducted to comprehensively characterize the strain’s main features. The results showed that the total size of the genome is 8,247,561 bp with 72.51% G + C content. Among a total of 7158 genes, 169 predicted RNA genes were identified including 67 transfer RNA (tRNA) genes, 18 ribosomal RNA (rRNA) genes and 84 small RNA (sRNA) genes, as well as 14 genomic islands were predicted. A total of 31 biosynthetic gene clusters were detected within the genome of *Streptomyces rochei* FE-3–1, and at least four of these gene clusters are associated with known potent antimicrobials. These findings provide a solid theoretical foundation for utilizing strain FE-3–1 in developing biofertilizers or biopesticides within the field of biotechnology.

## 1. Introduction

With the increasingly extensive and frequent use of antibiotics, the overuse of antibiotics has become a serious global health problem [1]. Subsequently, due to the increasingly serious problem of antimicrobial resistance, and the research and development of novel antibiotics have been becoming more urgent [2].

*Streptomyces* can survive in different habitats such as soil, plant bodies, volcano, desert, animal, limestone, sea water, and fresh water, etc [3]. *Streptomyces* strains have strong metabolic capacity and can better adapt to changing environments, and represent the biggest genus in the phylum of *Actinobacteria* [4]. It is a class of Gram-positive, high G + C content, and spore-forming aerobic *actinomycetes* with highly branched basal mycelia and gas mycelia, which is capable of metabolizing and synthesizing abundant secondary metabolites [5]. Several natural products have been derived from *actinomycetes* [6]. *Streptomyces* are the most significant producers of antibiotics among bacterial species. They also play a crucial role as agents for growth promotion and biocontrol, with their application in agriculture becoming increasingly prevalent [7,8].

While the genome of *Streptomyces* contains abundant gene clusters for secondary metabolites, its structure is complex and often experiences partial gene silencing, making it challenging to discover new active substances [9]. The analysis of genomes has revealed that *Streptomyces* species can harbor a range of 25–70 biosynthetic gene clusters [10]. Whole genome sequencing and genome mining can accurately predict the number and type of secondary metabolic gene clusters, the size, location, function and skeleton structure of coding genes, as well as metabolic regulation mode, which is conducive to the rapid mining of new metabolites [11]. Currently has 1290 *Streptomyces* genome published HTML (http://www.bacterio.net/streptomyces).

Numerous bioactive molecules have previously been isolated from *Streptomyces* species, exhibiting reported activities of secondary metabolites such as antifungal, antibacterial, anti-inflammatory, antitumor, and immunosuppressive effects [6]. Streptomycin and actinomycin were the first antibacterial compounds to be isolated from *Streptomyces* [12]. Currently, a variety of known antibiotics including streptomycin, tetracycline, erythromycin, chloramphenicol, lincomycin, kanamycin, clindamycin are derived from *Streptomyces* [13].

*Streptomyces* are the largest bacterial genus among the plant-associated *actinomycetes* isolated so far [14], and the roles of *Streptomyces* in the biological control of plant pathogens are also significant. Some metabolites of *Streptomyces* are active plant growth promotion [15]. Some *Streptomyces* can also produce enzymes that have extracellular cell wall degrading activity, thereby improving plant disease resistance [16,17].

Based on different *Streptomyces* strains, many commercial biocides, such as Actinovate (*Streptomyces lydicus*), Rhizovit (*Streptomyces* sp. DSMZ12424), and Mycostop (*Streptomyces griseoviridis*), have been developed for agricultural purposes [18–20]. Therefore, *Streptomyces* are important resources for agricultural biocides or biofertilizers.

In order to further understand the biological function and secondary metabolic potential of *Streptomyces rochei* FE-3–1, a genome-wide assay was performed and the complete genome sequence of the strain FE-3–1 was obtained. Gene prediction, functional annotation, secondary metabolic synthesis and comparative genome analysis were studied. This study provides a theoretical basis for further exploring its biocontrol potential and developing secondary metabolites that can be used for agricultural biological control.

## 2. Materials and methods

### 2.1. Isolation of *Streptomyces* strains

Gradient dilution separation was employed to isolate *Streptomyces* strains from rice rhizosphere soil samples [21]. Specifically, l g of soil sample was added into a flask containing 99 mL of sterile water, diluted at l:100 ratio, and shaken at 160 rpm for 30 min. After standing, the supernatants were diluted with sterile water into 10^−3^, 10^−4^, and 10^−5^ fold dilutions. Gause No.l agar medium containing antifungal K_2_Cr_2_O_7_ 75 μg/mL was spread with 200 μL of each soil dilution and incubated at 28°C for 5 ~ 7 days. The isolated single colonies were purified, cultured for 5 days, and transferred to Gause No.l agar medium for preservation.

### 2.2. Determination of anti-*Pyricularia oryzae* activity

The pathogenic fungi *Pyricularia oryzae* was inoculated onto PDA medium, and the fungal cake was prepared using a 6 mm hole punch and then inoculated at the center of the PDA plate. The strain FE-3–1 was streak-inoculated on two opposite edges from the center of the plate. The fungal cake of *Pyricularia oryzae* was separately inoculated as a control. Three replicates were conducted for each treatment. The colony radius (from the edge of the cake to the edge of pathogenic fungi) was determined by the cross-crossing method using vernier calipers, and inhibition rate was calculated. Relative inhibition rate (%) = (control pathogen colony radius – pathogen colony radius on the plate inoculated with antagonistic strain FE-3–1)/control pathogen colony radius ×100%.

### 2.3. Identification of the strain FE-3–1

The strain FE-3–1 was inoculated and purified in Gause No.1 agar medium, followed by cultivation at 28°C for 5 ~ 7 days to observe colony characteristics and color. Spore morphology and mycelium were also examined via optical microscopy. Total DNA was extracted from the purified FE-3–1 strain using QIAamp DNA Mini Kit (Qiagen, CA, USA) according to the manufacturer’s protocol, and the 16S rDNA gene sequence was amplified via PCR with universal primers 27F and 1492R. The resulting DNA sequences were aligned using ClustalW, and a phylogenetic tree was constructed using MEGA 6.0 [22].

### 2.4. Genome assembly, scaffolding, and annotation

The whole genome sequence of the strain FE-3–1 was obtained through Pacbio and Illumina Hiseq×10 platforms with approximately 100-fold coverage in both platforms. Genomic DNA was randomly fragmented using Covaris or Bioruptor method, followed by sequencing adaptor ligation according to the manufacturer’s instructions for Illumina Paired-End sequencing library preparation. The prepared libraries and genome were sequenced. The resulting reads were de novo assembled via SOAPdenovo v1.05. The genome was annotated by the NCBI Pro-karyotic Genome Annotation Pipeline, and genes were identified by GeneMarkS^+^.

### 2.5. Screening genes related to beneficial traits

Biosynthetic gene clusters responsible for synthesizing active secondary metabolites were predicted in the genome of the strain FE-3–1 using antiSMASH bacterial version 7.0 (http://antismash.secondarymetabolites.org/). Additionally, Prokka- annotated genes were screened for plant growth promotion traits, such as phosphorus solubilization, potassium solubilization, nitrogen assimilation, siderophore production, plant hormone production as well as biocatalysts including protease, lipase, chitinase, and catalase production. Furthermore, heavy metal resistance genes were also manually screened.

### 2.6. Gene functional category

Functional enrichment analysis classified core gene families into different biological functions based on COG/GO/KEGG databases. The numbers of corresponding proteins were computed for each term of COG/GO/KEGG, and the resulting results were visualized by GraphPad Prism 7.0.

### 2.7. Pan-genome and comparative genomic analysis

A pan-genome analysis involving 9 *Streptomyces*-related species’ genomes was manipulated using Roary Pan-genome Pipeline, and regression analysis of core gene cluster curves was conducted by weighted least square regression.

### 2.8. Statistical analysis

Means and standard errors of the data were calculated using Excel 2010 (Microsoft Corporation, USA). All data collected were statistically analyzed according to Duncan’s multiple range test (P = 0.05).

## 3. Results

### 3.1. Isolation and identification of FE-3–1

**Fig 1:**
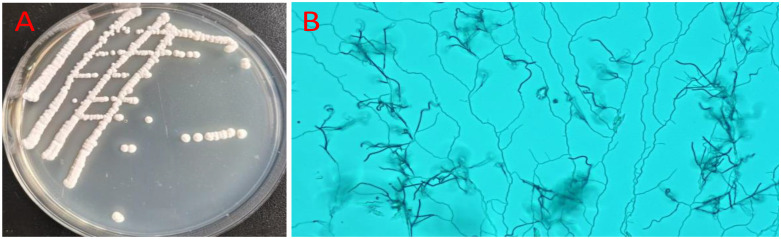
The colony morphology of *Streptomyces rochei* FE-3-1 (A). The morphology of hyphae of *Streptomyces rochei* FE-3-1 observed under 100-fold optical microscopy (B).

**Fig 2:**
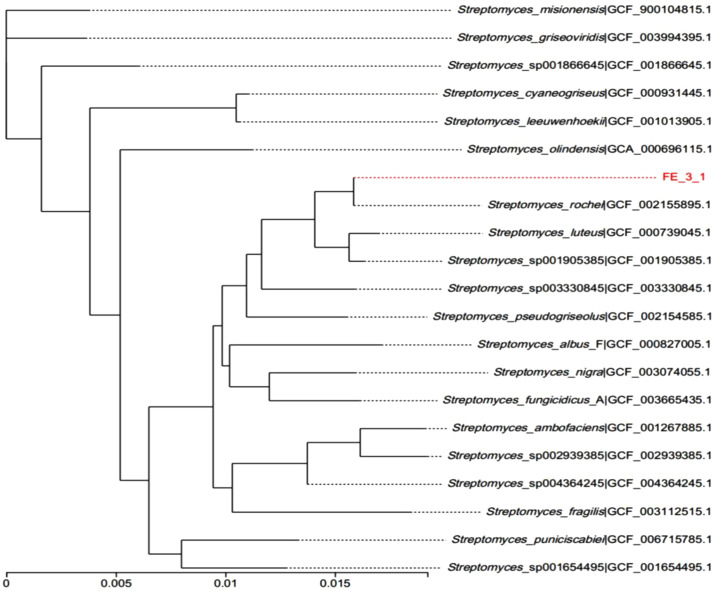
The phylogenetic tree of *Streptomyces rochei* FE-3-1 based on the 16S rDNA gene sequence.

The colonies of the strain FE-3–1 are nearly round, dry, with protrusions in the middle, and opaque with a regular slick edge on Gause No.1 agar plates (Fig 1A). The spore mass is gray and the basal mycelium is orange. The strain FE-3–1 is Gram-positive and aerobic, with vigorously growing aerobic hyphae that have more branches (Fig 1B). The basic characteristics and classification of the strain FE-3–1 are shown in S1 Table in S1 File. Based on the 16S rDNA gene sequences, the phylogenetic tree of the strain FE-3–1 was constructed using neighbor-joining method, and phylogenetic analysis placed the strain FE-3–1 as most closely related to *Streptomyces rochei* NRRL B-2410 (Fig 2).

### 3.2. Anti-*Pyricularia oryzae* activity analysis

**Fig 3:**
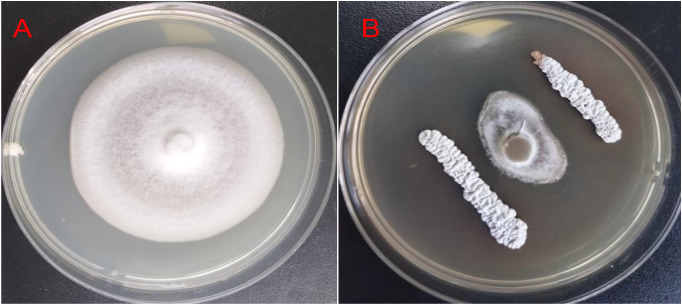
The inhibitory effect of strain FE-3-1 on *Pyricularia oryzae.*

The strain FE-3–1 exhibited inhibitory effect on plant pathogen *Pyricularia oryzae* (Fig 3), indicating its potential as a source of anti-*Pyricularia oryzae* compound. The results demonstrated that the colony radius of pathogenic fungi *Pyricularia oryzae* on the plate inoculated with antagonistic strain FE-3–1 was 8.00 ± 0.035 mm, whereas that of control pathogen fungi was 30.00 ± 0.046 mm, resulting in an inhibition rate of 73.33 ± 0.079%.

### 3.3. Genome sequencing, annotation and features

**Fig 4:**
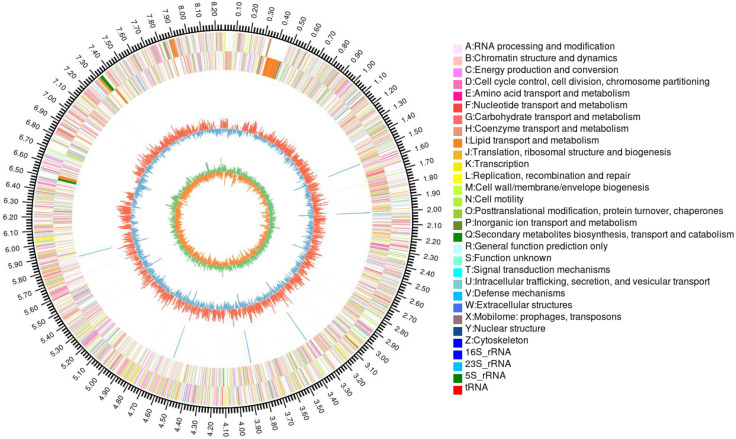
A graphical circular map of *Streptomyces rochei* FE-3-1. From outside to center, the outermost ring shows the marker of the genome size; rings 2, 3 show protein-coding genes colored by COG categories on forward/reverse strand; rings 4 shows rRNA and tRNA; ring 5 shows G + C % content plot; the innermost circle is the GC-Skew.

**Fig 5:**
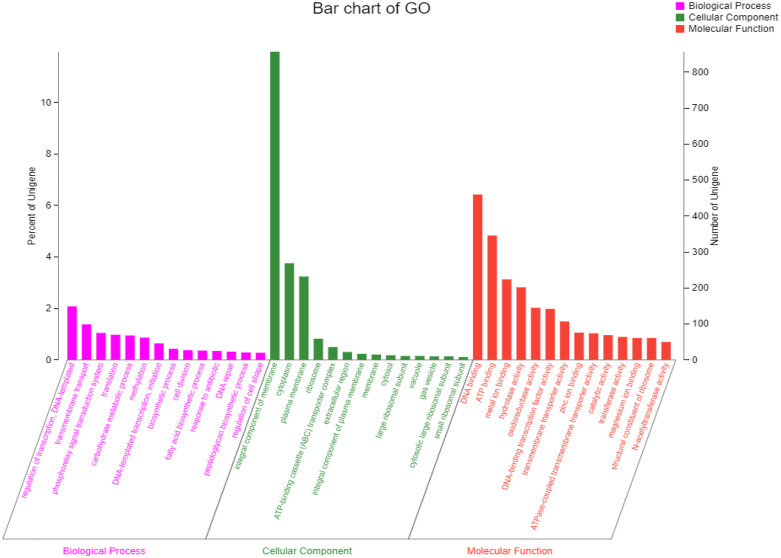
GO functional classification map of *Streptomyces rochei* FE-3-1.

**Fig 6:**
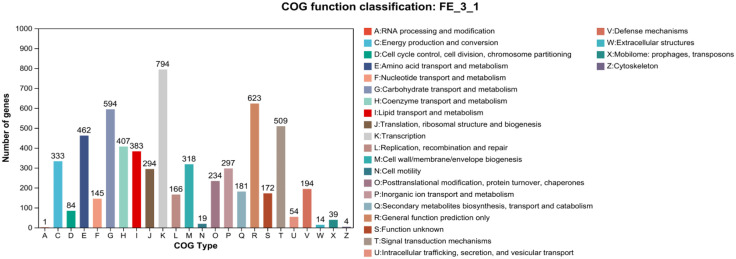
COG functional classification map of *Streptomyces rochei* FE-3-1.

**Fig 7:**
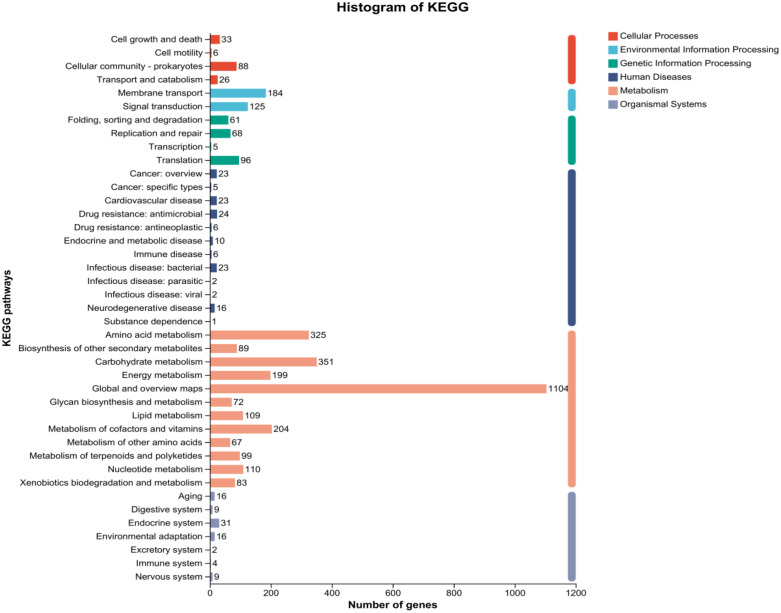
KEGG classification of metabolic pathways map of *Streptomyces rochei* FE-3-1.

The strain was selected for sequencing primarily due to its anti-*Pyricularia oryzae* activity. Genome was sequenced by Shanghai Majorbio Bio-pharm Technology Co., Ltd (Shanghai, China). The project information is summarized in S2 Table in S1 File. A standard shotgun library generated 341255 reads with an average length of 4546.05 bp. The total size of the genome is 8,247,561 bp with a G + C content of 72.51% (Fig 4). The genome properties and statistics are summarized in Table 1. Among the total of 7158 genes, there were 169 predicted RNA genes including 67 transfer RNA (tRNA) genes, 18 ribosomal RNA (rRNA) genes and 84 small RNA (sRNA) genes, as well as 14 genomic islands (S3 Table in S1 File). Functional annotation of genes from the strain FE-3–1 was performed using GO (Fig 5), COG (Fig 6) and KEGG (Fig 7) databases which revealed active transcription, metabolism, signal transduction, secondary metabolites biosynthesis and membrane transport activities (Fig 5). Additionally, 5571 (77.83%) genes are distributed into COG functional categories (Fig 6). The 2846 annotated gene products are involved in 254 pathways, including metabolism (143 pathways), cellular processes (13 pathways), genetic information processing (16 pathways), organismal systems (30 pathways), human diseases (30 pathways), and environmental information processing (13 pathways).

**Table 1:** The genome properties and statistics of *Streptomyces rochei* FE-3-1.

Attributes	Values
Genome size (bp)	8247561
CDS No.	7158
G + C Content(%)	72.51
tRNA No.	67
Type of tRNAs No.	20
rRNA No.	18
Gene No.	7158
Gene total length(bp)	7280481
Gene average length(bp)	1017.11
Gene density(kb)	0.87
GC content in gene region(%)	72.74
Gene Len/Genome(%)	88.27
Intergenetic region length(bp)	967080
GC content in intergenetic region(%)	70.81
Intergenetic length/Genome(%)	11.73
Total reads num	341255
Average length of reads(bp)	4546.05
CRISPR-Cas No.	17
Total lengthof tandem repeat(bp)	70328
Tandem repeat/Genome(%)	0.97
Genes No. of Cellular Component	1354
Genes No. of Molecular Function	2993
Genes No. of Biological Process	1460
Genes assigned to COGs	5571
Genes with Pfam domains	5889

### 3.4. Identification of heavy metal resistance genes

According to the results of genomic annotation, the strain FE-3–1 harbors multiple putative functional proteins associated with different heavy metals (Cadmium, Arsenic and Copper) or metal (Zinc), and putative functions including transporters, P-type ATPase and resistance proteins, and so on (Table 2). The strain exhibits resistance to a variety of heavy metals or metal, enabling it to effectively mitigate the impact of heavy metal or metal pollution, particularly in environments characterized by combined cadmium and arsenic contamination.

**Table 2:** Putative proteins involved in metal resistance.

Metals	Gene name	Gene ID	Putative function	BLASTP analysis
Cadmium	copA	gene2642	Cadmium-translocating P-type ATPase	WP_199577713.1
	ctpJ	gene6062	Cadmium-translocating P-type ATPase	WP_185912699.1
Arsenic	arsB	gene0746	Arsenic transporter	WP_136235124.1
Zinc	znuA	gene2435	Zinc ABC transporter substrate-binding protein	WP_127433667.1
	ftsH	gene5105	ATP-dependent zinc metalloprotease	WP_019328772.1
Copper	ycnJ	gene3545	Copper resistance protein	WP_127433257.1

### 3.5. Genes related to plant growth promotion traits

According to Prokka annotations, the genome of *Streptomyces rochei* FE-3–1 harbors numerous genes associated with promoting plant growth, including those involved in phosphate solubilization, potassium solubilization, nitrogen assimilation and siderophore production (Table 3). Additionally, the presence of genes responsible for chitinase production and related lyases, such as protease and lipase, suggests potent antifungal properties, and antifungal activity of the strain has been confirmed in Fig 1. Furthermore, the peroxidase/catalase-related genes were also detected within the genome of *Streptomyces rochei* FE-3–1 and the production of peroxidase and catalase by strain FE-3–1 may be an important mechanism of plant resistance to oxidative stress.

**Table 3:** Some genes related to the plant growth promotion within *Streptomyces rochei* FE-3-1 according to Prokka annotations.

Traits	Genes	Products
Nitrogen assimilation	nirB, nirD	Nitrite reductase
	narK,	Nitrate/nitrite transporter
	narI, narJ, narH, narG	Nitrate reductase
Phosphate solubilization	*rsb*U_P, *prp*C	Protein phosphatase
	phoD	Alkaline phosphatase D
	rhnA-cobC	RNase H/acid phosphatase
	maf	Nucleotide pyrophosphatase
	ppa	Inorganic diphosphatase
	gpmB	Histidine phosphatase
Potassium solubilization	kdpA, kdpB, kdpC	Potassium-transporting ATPase
	trkA	Potassium uptake protein
	cvrA	Potassium/proton antiporter
Iron sequestration	fhuD	Iron-siderophore ABC transporter substrate-binding protein
	–	Siderophore-interacting protein
Phytohormone synthesis	trpC	Indole-3-glycerol phosphate synthase
Biocatalyst	snpA	Zinc-dependent metalloprotease
	ftsH, htpX	Zinc metalloprotease
	hyaD	Hydrogenase maturation protease
	–	Lipase
	plc	Phospholipase
	pnbA	Carboxylesterase/lipase
	–	Chitinase
	amyA	Alpha-amylase
	katE	Catalase
	katG	Catalase/peroxidase

### 3.6. Prediction of secondary metabolites-related gene clusters

**Fig 8:**
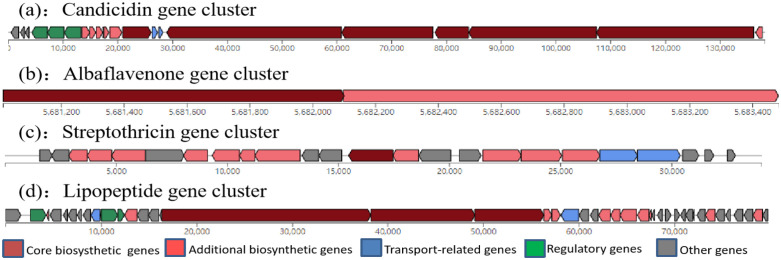
Similarity to known antimicrobial metabolites biosynthetic gene clusters detected with antiSMASH in the genome of *Streptomyces rochei* FE-3-1: (A) Candicidin gene cluster; (B) Albaflavenone gene cluster; (C) Streptothricin gene cluster. The characteristics of the clusters and gene types are indicated in the box.

A total of 31 biosynthetic gene clusters were identified within the genome of *Streptomyces rochei* FE-3–1 via antiSMASH bacterial version 7.0 (Table 4). Four of these biosynthetic gene clusters exhibited high similarity to known metabolites with antifungal and antibacterial properties such as candicidin, streptothricin, lipopeptide, and albaflavenone (Fig 8). Some gene clusters were also related to active metabolites such as isorenieratene, desferrioxamine, hopene, ectoine, sapB, fluostatins, 7-prenylisatin, melanin, borrelidin and geosmin. Some gene clusters showed limited or no similarity to known metabolites. These types of gene clusters included terpene, indole, ectoine, melanin, arylpolyene, T2PKS, NRPS, lanthipeptides, and siderophores.

**Table 4:** The biosynthetic gene clusters detected with AntiSMASH within the genome of *Streptomyces rochei* FE-3-1.

Cluster ID	Start	End	MIBiG accession	BGC Type	Most Similar Known Metabolies	% Identity	Gene No.
Cluster1	22348	106504	BGC0001596	T2PKS	fluostatins M-Q	67	76
Cluster2	175975	195904	BGC0001294	indole	7-prenylisatin	100	12
Cluster3	199327	223208	BGC0000664	terpene	Isorenieratene	100	21
Cluster4	283217	491949	BGC0000034	NRPS	Candicidin	95	67
Cluster5	511227	531536	BGC0000242	terpene	lysolipin I	4	21
Cluster6	795716	816838	BGC0001483	indole	5-isoprenylindole-3-carboxylate β-D-glycosyl ester	33	23
Cluster7	875531	898962	BGC0000633	terpene	carotenoid	54	20
Cluster8	1092239	1151522	BGC0000432	NRPS	streptothricin	100	54
Cluster9	1385222	1426329	BGC0001065	T3PKS	herboxidiene	8	39
Cluster10	2084186	2094585	BGC0000853	ectoine	ectoine	100	10
Cluster11	2965862	2976471	BGC0000910	melanin	melanin	100	12
Cluster12	3063027	3073950	BGC0000940	siderophore	desferrioxamin B/ desferrioxamine E	83	9
Cluster13	4094137	4137242	BGC0000227	NRPS-like	granaticin	8	35
Cluster14	4236271	4277228	BGC0000914	PKS-like	methylenomycin A	9	44
Cluster15	4375634	4395269	BGC0000551	lanthipeptide	SapB	75	16
Cluster16	4605927	4668548	BGC0000806	NRPS	phosphonoglycans	5	61
Cluster17	5327755	5348378	BGC0000660	terpene	albaflavenone	100	20
Cluster18	5386479	5455743	BGC0000271	T2PKS	spore pigment	66	62
Cluster19	5911783	5921934	–	siderophore	–	–	7
Cluster20	6178528	6188937	–	bacteriocin	–	–	10
Cluster21	6205131	6224912	BGC0001181	terpene	geosmin	100	18
Cluster22	6386962	6400136	BGC0001732	siderophore	paulomycin	9	12
Cluster23	6461777	6551885	BGC0001370	arylpolyene	lipopeptide 8D1-1/ lipopeptide 8D1-2	86	52
Cluster24	6777423	6802165	–	lanthipeptide	–	–	28
Cluster25	7154187	7180115	BGC0000663	terpene	hopene	100	24
Cluster26	7285725	7378488	BGC0001785	T1PKS	streptovaricin	31	34
Cluster27	7508419	7529451	–	terpene	–	–	20
Cluster28	7540869	7551085	BGC0000518	bacteriocin	informatipeptin	42	5
Cluster29	7810082	7922797	BGC0000031	NRPS	borrelidin	88	66
Cluster30	8028118	8052713	–	lanthipeptide	–	–	20
Cluster31	8193271	8247561	BGC0000236	lassopeptide	kinamycin	31	57

### 3.7. Prediction of anti-*Pyricularia oryzae* activity

The strain FE-3–1 exhibited some antagonistic activity against *Pyricularia oryzae*, suggesting its potential as a biological control agent. Among the 31 gene clusters responsible for secondary metabolite production, the lipopeptides 8D1-1 and 8D1-2 from the NRPS/PKS gene cluster may play a major role in antagonizing *Pyricularia oryzae*. In the follow-up study, we will carry out the isolation, identification and function study of lipopeptides 8D1-1 and 8D1-2.

### 3.8. Molecular phylogenetic analysis of FE-3–1 and 8 other strains

**Fig 9:**
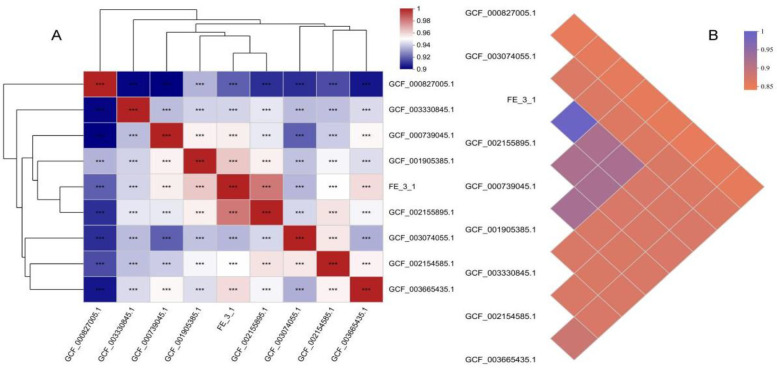
Molecular phylogenetic analysis of FE-3-1 and 8 other strains of *Streptomyces rochei.* (A) Correlation analysis of of 9 *Streptomyces* strains based on 16S rDNA sequence. 0.01 < p ≤ 0.05 *, 0.001 < p ≤ 0.01 **, p ≤ 0.001 ***. (B) ANI triangular heatmap of 9 *Streptomyces* strains. On the left is the sample name, and the squares of different colors indicate the average nucleotide similarity between the samples.

**Fig 10:**
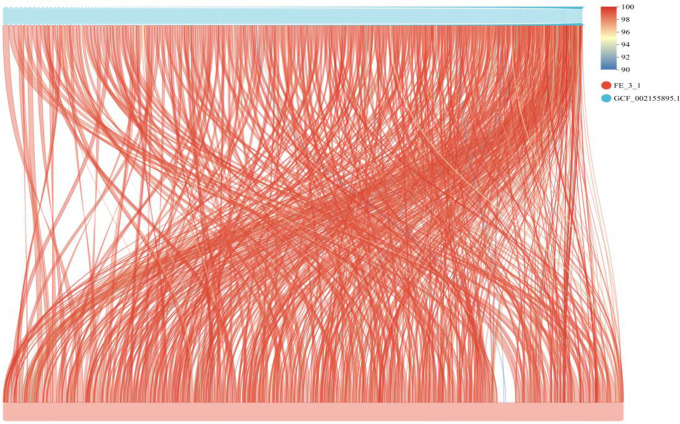
Mauve synteny alignment analysis of the genome sequences of FE-3-1 and 8 other strains of *Streptomyces rochei.* The upper and lower bars of color blocks represent two different genomes, and the regions of the two genomes are connected by lines, the color of which represents the degree of collinearity.

To further clarify the taxonomic status of strain FE-3–1 at the species level, we compared its genome correlation index with that of 8 closely related model strains (S4 and S5 Tables in S1 File). Correlation analysis indicated a stronger correlation between different samples, with a highly significant difference (Fig 9A). The results also revealed that both the average nucleotide identity (ANI) and average amino acid identity (AAI) of strain FE-3–1 and *Streptomyces rochei* NRRL B-2410 were higher than the threshold values for species classification (ANI > 95%−96%, AAI > 95%−96%) (Fig 9B). Additionally, the genome G + C content of strain FE-3–1 and *Streptomyces rochei* NRRL B-2410 was found to be 72.51% and 72.50%, respectively, with a difference within the variation range of the same species (<1%). Furthermore, a syntenic analysis of FE-3–1 with *Streptomyces rochei* NRRL B-2410 genomes using Progressive Mauve Align showed a highly conserved synteny between them (Fig 10). Taking into consideration these genomic findings along with its morphological characteristics, we preliminarily identified strain FE-3–1 as belonging to *Streptomyces rochei*.

### 3.9. Features of the core and pan-genomes

In order to better understand the genetic diversity of *Streptomyces*, we selected 8 of *Streptomyces* strains that are closely related to *Streptomyces rochei* FE-3–1 (Fig 9A), and studied the relationship between the size of the pan-genome and core genome and the number of genomes. With the addition of new strains, the number of core orthologous gene clusters consistently decreased, while the size of the pan-genome gradually increased (S1 and S2 Figs in S1 File). The gene content of in *Streptomyces rochei* FE-3–1 was compared with 8 other related reference strains. The comparison results showed that a total of 17,816 gene families were found in the 9 genomes, of which 2,920 genes constituted the core genome. The functional classes of the core gene families were further determined by homologous group (COG) attribution among all closely related species. The results showed that the core gene families were unevenly distributed across functional classes (Fig 6).

### 3.10. Comparative genomic analysis

**Fig 11:**
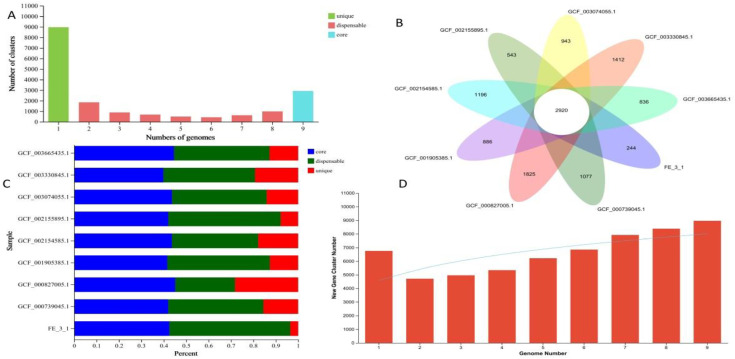
Comparative genomic analysis of FE-3-1 and 8 other strains of *Streptomyces rochei.* (A) Statistics of homologous genes in the genomes; (B) Flower plot displaying the numerous core orthologous gene clusters and specific orthologous gene clusters in 9 strains of *Streptomyces rochei.* Each petal represented a strain. The number of core orthologous gene clusters in shown in the center. The number in non-overlapping portions showed the numbers of strain-specific orthologous gene clusters. The strain name is located beside the petal; (C) Distribution of orthologous gene clusters in different samples; (D) Histogram and graph of the number of new gene clusters as a function of the number of genomes. A new gene is the number of gene clusters that are added when a genome is added for pan-genome analysis.

In this study, the amino acid sequences of nine *Streptomyces* species involved were aligned using the OrthoMC, and a specific threshold (E-Value: 1e-5, Percent Identity Cutoff: 0, Markov Inflation Index: 1.5) was selected for similarity clustering in order to obtain homologous genes. A total of 8962 orthologous gene clusters were identified, with 2920 gene clusters being core orthologous gene clusters among the nine *Streptomyces* strains, and the number of homologous gene clusters co-existing in 2 or more samples was smaller than that in all samples (Fig 11A).Venn diagrams were also used to intuitively display core and unique homologous genes between species. Among these, 244 clusters were found to be unique to *Streptomyces rochei* FE-3–1 and 543 clusters were unique to *Streptomyces rochei* NRRL B-2410 (GCF_002155895.1) (Fig 11B). The proportion of core, dispensable, and unique orthologous gene clusters varied across different samples’ genomes (Fig 11C), and the number of new gene clusters gradually increased with the growing number of genomes (Fig 11D).

## 4. Discussion

With the rapid advancement of genome sequencing technology, large-scale genome sequencing continues to uncover the diverse natural products present in microbial resources. The biosynthetic potential of microorganisms has been significantly underestimated, ushering in a new era of microbial natural product mining post-genomics [23]. Genome mining utilizes bioinformatics analysis tools to predict secondary metabolic gene clusters for targeted product discovery, playing a crucial role in drug development [24]. Many microbial metabolites remain undiscovered through traditional low-throughput approaches because of the association of natural products with cryptic genes, which frequently demand external stimulation for microbes to generate. Genome mining expedites the identification of those cryptic and uncharacterized biosynthetic gene clusters accountable for synthesizing natural products, which are likely novel [24]. Furthermore, genomic information is invaluable for studying strain evolution, response mechanisms, and environmental adaptation [25].

Rhizobacteria play a vital role in promoting plant growth under various environmental conditions and also contribute to biocontrol by inhibiting plant pathogens through the production of secondary metabolites [26,27]. *Streptomyces* species are known for their rich source of bioactive substances [10]. In this study, *Streptomyces rochei* FE-3–1 isolated from rice rhizosphere soil exhibited inhibitory effect on plant pathogen *Pyricularia oryzae* and holds potential for biocontrol applications as a rhizosphere *Streptomyces*.

Of all published related genomes of *Streptomyces rochei* FE-3–1, *Streptomyces rochei* NRRL B-2410 (GCF_002155895.1) shows the lowest genomic distance, and the strain is also isolated from soil and is the typical strain of *Streptomyces rochei*. The G + C content of *Streptomyces rochei* NRRL B-2410 was 72.5%, the number of genes was 7276, and the protein expression genes were 6727. Most of the members of the *Streptomyces rochei* group were reported as antibiotic-producing strains [28,29]. The genome of *Streptomyces rochei* NRRL B-2410 are understudied.

Comparative genomic analysis of nine distinct *Streptomyces* strains demonstrated that 244 unique gene clusters were specific to the strain FE-3–1. Among these unique homologous genes, such as nitrite reductase [EC:1.7.2.1], pyruvate carboxylase [EC:6.4.1.1], and polyphosphate kinase [EC:2.7.4.34], etc. The enzyme proteins encoded by these gene clusters play crucial roles in promoting growth, synthesizing secondary metabolites and tolerating external environmental stress. Moreover, these unique homologous genes may contribute to the strain’s distinctive capabilities.

**Fig 12:**
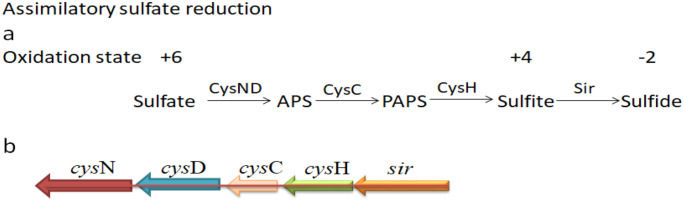
Putative sulfur metabolism pathway and assimilatory sulfate reduction genes in *Streptomyces rochei* FE-3-1. a Putative sulfur metabolism pathway. b Assimilatory sulfate reduction genes in *Streptomyces rochei* FE-3-1.

**Fig 13:**
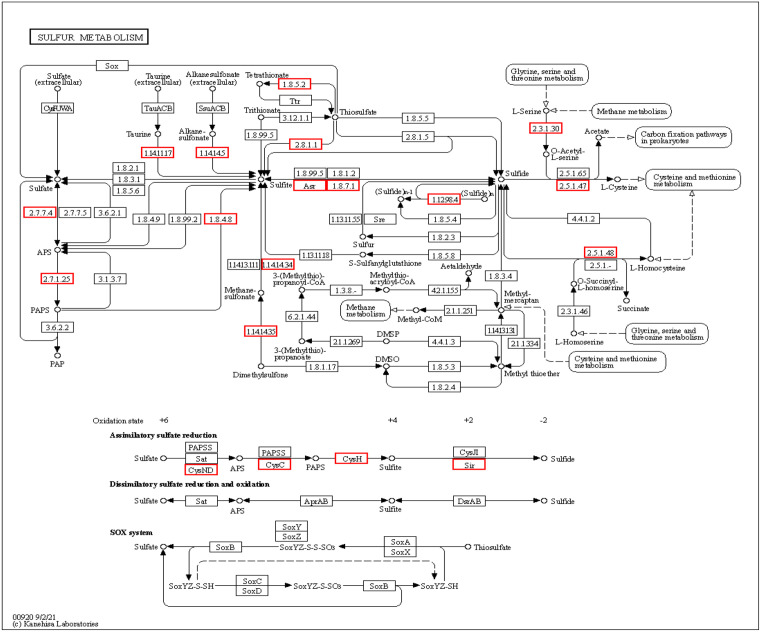
The sulfur metabolism is illustrated using a KEGG pathway diagram. The proteins highlighted in red boxes were identified in strain FE-3-1. Numbers indicate E.C. numbers of enzymes.

The KEGG pathways of *Streptomyces rochei* FE-3–1 show some genes that are beneficial to plants (Table 3). For example, reducing nitrate nitrogen in soil to ammonia is a beneficial ability for plants. It converts nitrate and nitrite compounds into ammonia compounds, which contributes to the availability of nitrogen for plants [30]. The strain can produce hydrogen sulfide through sulfur metabolism pathway (Table 5, Fig 12), which can combine with free heavy metal ions in soil to form residual sulfide, increase plant stress resistance and reduce the absorption and transport of heavy metals by plants [31]. Hydrogen sulfide can also act as a signaling molecule, promoting seed germination and seedling growth [32]. The sulfur metabolism pathway based on KEGG is illustrated in Fig 13. The strain has the ability to nitrogen assimilation, phosphate solubilization and potassium solubilization. By producing enzymes, the phosphorus that is difficult for plants to absorb and utilize is converted into absorbable and available phosphorus [33], and the insoluble potassium that plants cannot absorb is converted into soluble potassium [34]. By improving the utilization rate of nitrogen, phosphorus and potassium by plants, the strain promotes plant metabolism and regulates plant growth and reproduction. The strain FE-3–1 might function as a biocontrol agent through diverse mechanisms. The enhancement of plant vigor in the face of various biotic stresses, diseases, and pests is also regulated by an adequate supply of nitrogen, phosphate, and potassium.

**Table 5:** Putative proteins involved in sulfate reduction.

Gene name	Gene ID	Putative function	BLASTP analysis
cysN	gene5529	Sulfate adenylyltransferase subunit	WP_019326036.1
cysD	gene5530	Sulfate adenylyltransferase	WP_019326037.1
cysC	gene5531	Adenylyl-sulfate kinase	PVD05672.1
cysH	gene5532	Phosphoadenylyl-sulfate reductase	WP_125772742.1
sirA	gene5534	Sulfite reductase	WP_109204242.1

Subsequent genomic analysis identified three siderophore gene clusters 12, 19, and 22 in the genome of *Streptomyces rochei* FE-3–1. Cluster 12 shows high similarity (83%) to the biosynthetic gene cluster of desferrioxamin B and desferrioxamine E in *Streptomyces coelicolor* A3(2) and contains 9 necessary genes responsible for the desferrioxamin B and desferrioxamine E biosynthesis. The siderophore produced by the strain can chelate iron ions in the environment, convert it into a form available to organisms, and promote plant growth. The siderophore produced by rhizosphere bacteria can also compete with plant pathogens for iron ions, thus inhibiting their growth and improving plant disease resistance. The siderophore can also chelate many harmful metal ions, reducing the damage of toxic metal ions to plants [35].

In order to destroy the components of cell wall of pathogenic fungal, the biosynthetic gene clusters of many functional microorganisms can produce hydrolytic enzymes, such as protease, lipase, amylase and chitinase, which is also an important mechanism for functional microorganisms to control plant pathogens [36]. Indeed, *Streptomyces rochei* FE-3–1 exhibited the potential to inhibit the growth of pathogenic fungus on agar plate, which was further confirmed and identified three protease, two lipase, two catalase, one chitinase and one amylase genes via genome mining in the genome of *Streptomyces rochei* FE-3–1 (Table 3).

The soil heavy metal pollution constitutes a crucial limiting factor for the survival of the strain in the environment. Bacteria have evolved several mechanisms to resist and remediate heavy metals for their own survival [31]. Certain bacterial metabolic mechanisms specifically exist to immobilize and reduce the bioavailability of heavy metals. Immobilization can be accomplished through sorption to cell components and exopolymers, transport, intracellular sequestration, or precipitation of metal ions as insoluble organic and inorganic compounds [37]. Based on the outcomes of genome annotation, the strain FE-3–1 possesses multiple putative functional proteins related to various heavy metals. These resistances to heavy metals enhance the strain FE-3–1’s capability to survive in extreme environments and lay a foundation for its colonization of the rhizosphere and promotion of plant growth.

The antiSMASH results showed that there were 31 biosynthetic gene clusters. The number of NRPS and terpene-type biosynthetic gene clusters was the largest. PKS is significantly present in both terrestrial and aquatic environments and is closely related to the biosynthesis of antimicrobial metabolites in *actinomycetes*, especially *Streptomyces* [38]. Five of PKS and PKS-like gene clusters were found in the genome of *Streptomyces rochei* FE-3–1. At least three of the gene clusters detected in the genome of *Streptomyces rochei* FE-3–1 are associated with currently known potent antimicrobials. Streptothricin is an N-glucoside antibiotic, which exists in different *Streptomyces* and is one of the earliest antibiotics isolated from *Streptomyces* and has broad-spectrum activity against both Gram-positive, Gram-negative bacteria and pathogenic fungi. It inhibits protein biosynthesis in prokaryotic cells by suppressing polypeptide synthesis through ribosome interactions [39]. The candicidin complex was initially isolated from *Streptomyces griseus* IMRU3570. It demonstrates a potent inhibitory activity against fungi. The candicidin complex was regarded as the prototype of aromatic polyene macrolides. Similar to other polyketide polyenes, candicidin can interact with ergosterol existing in the membranes of fungi to form a transmembrane channel, resulting in K^+^ leakage and causing cell death [40]. Albaflavenone was initially isolated from *Streptomyces albidoflavus* and is recognized for exhibiting antibacterial properties. The biosynthetic pathway of albaflavenone has recently been elucidated in *Streptomyces coelicolor* A3(2) [41]. Lipopeptide antibiotics represent the most recent addition to the antibiotic family, with molecular weights typically ranging from 1000−1600 Da. They are synthesized via a non-ribosomal multi-enzyme biosynthesis pathway [42]. Surfactin, iturin, and fengycin are exemplary lipid peptides known for their intricate effects on plant pathogenic fungi [43]. Existing literature also contains relevant reports on lipopeptides antagonizing Pyricularia oryzae [44–46]. These results may explain the extensive and effective bacteriostatic activity of *Streptomyces rochei* FE-3–1. Other biosynthetic gene clusters are associated with other active metabolites, including isoprene, which has antioxidant effects. Ectoine, which is resistant to salt stress. Siderophore, which has strong chelating ability to iron. melanin, which can resist environmental stress, and borrelidin, which has herbicidal activity.

Numerous commercial biocontrol agents derived from diverse *Streptomyces* strains, including Actinovate, Rhizovit, and Mycostop, exhibit resistance to a wide range of pathogens (Table 6) [19]. While this study demonstrates the potent *in vitro* antagonism of *Streptomyces rochei* FE-3–1 metabolites against *Pyricularia oryzae*, a direct quantitative comparison of inhibition rates to commercial products like Actinovate (*Streptomyces lydicus*), Rhizovit (*Streptomyces* sp.), or Mycostop (*Streptomyces griseoviridis*) requires further bioassay against a common pathogen panel. However, the genomic analysis reveals FE-3–1 possesses significant potential comparable to these commercially exploited strains. Its genome harbors 31 biosynthetic gene clusters, at least four linked to known potent antimicrobials. This genomic richness suggests FE-3–1 has the inherent capacity to produce a diverse arsenal of bioactive compounds, potentially explaining its observed activity and supporting its potential to resist a wide range of plant pathogens. This preliminary genomic evidence positions FE-3–1 as a strong candidate for development into novel biofertilizers or biopesticides, warranting further exploration of its metabolite production.

**Table 6:** Commercialized *streptomyces* based biopesticides [19].

Biocontrol agent(s)	Active ingredient	Target pathogen(s)	Biocontrol mechanism	Country
Actinovate	streptomyces lydicus WYEC108	Fusarium spp., Rhizoctonia spp., Pythium spp., Phytophthora spp., Erisiphe spp., Sphaeroteca spp., Laveillula spp., Sclerotinia spp.	Antibiosis and hyperparasitism	European Union
Rhizovit	Streptomyces rimosus	Pythium spp., Fusarium spp., Phomopsis spp., Phytophthora spp., R. solani, A. brassicola, Botrytis spp., Fusarium spp.	Antibiosis	–
Mycostop	Streptomyces griseoviridis K61	Ceratocystis radicicola, Alternaria spp., Rhizoctonia solani, Fusarium spp., Phytophthora spp., Pythium spp.	Competition, hyperparasitism, and antibiosis	Canada

## 5. Conclusions

This study isolated a *Streptomyces* strain, FE-3–1, from the rice rhizosphere, demonstrating significant inhibitory activity against the plant pathogen *Pyricularia oryzae*. Morphological and phylogenetic analysis identified the strain as *Streptomyces rochei*. Whole genome sequencing revealed a substantial biosynthetic potential, anchored by 31 biosynthetic gene clusters including at least four associated with known antimicrobials. This high-quality genome sequence provides a crucial resource for comparative actinobacterial genomics. Genome mining confirmed the strain’s strong capacity for secondary metabolite production, highlighting its value as a source of bioactive compounds. Collectively, these findings provide a robust theoretical foundation for developing *Streptomyces rochei* FE-3–1 into effective biofertilizers or biopesticides. Furthermore, the genomic insights facilitate the targeted mining of related, underexplored actinobacteria, paving the way for discovering next-generation biocontrol strains with novel or enhanced modes of action against diverse plant pathogens.

## Supporting information

S1 File Supplementary materials. (DOC)

## Abbreviations

KEGG: Kyoto Encyclopedia of Genes and Genomes
NCBI: National Center for Biotechnology Information
COG: Cluster of Orthologous Groups of Proteins
GO: Gene Ontology
rRNA: ribosomal RNA
tRNA: transfer RNA
sRNA: small RNA.
