# Supplementary material for: Isolation, identification, and whole-genome sequencing of Streptomyces rochei FE-3-1 against Pyricularia oryzae
Source: PLoS One. 2025 Oct 29;20(10):e0331386. doi: 10.1371/journal.pone.0331386 (PMC12571248; doi:10.1371/journal.pone.0331386)
Supplement: S1 File — (DOC) [file pone.0331386.s001.doc]

*Title page-Supporting materials*

**Isolation, identification, and whole-genome**

**sequencing of *Streptomyces rochei* FE-3-1 against *Pyricularia oryzae***

Dongxia Du^1,3^, Zhuo Yi^2^,*, Shiping Shan^1,3^,*, Shuaishuai Gao^1^, Mengyuan Yu^1^, Bin Wang^1,3^

1 *Hunan Institute of Microbiology, Changsha, Hunan, 410009, China*

*2 Yiyang Open University, Yiyang, Hunan , 413000, China*

3 *Hunan Engineering and Technology Research Center of Agricultural Microbiology Application*, *Changsha, Hunan, 410009, China*

*Author for correspondence:

Zhuo Yi ([yizhuo_2008@126.com](mailto:yizhuo_2008@126.com)),

Current Address: Yiyang Open University, Yiyang, Hunan , 413000, China

Shiping Shan ([ssp312@hotmail.com](mailto:ssp312@hotmail.com))

Curent Address: Hunan Institute of Microbiology, Changsha, Hunan, 410009, China.

Phone: (+86) 731 88858945.

Fax: (+86) 731 85261072.

The supporting information has 8 pages including 5 tables and 2 figures.

**S1 Table**

S1 Table Classification and general features of *Streptomyces rochei* FE-3-1

| Property | Term |
| --- | --- |
| Classification | Domain *Bacteria* |
|  | Phylum *Actinobacteria* |
|  | Class *Actinobacteria* |
|  | Order *Streptomycetales* |
|  | Family *Streptomycetaceae* |
|  | Genus *Streptomyces* |
|  | Species *rochei* |
| Gram stain | Gram-positive |
| Cell shape | Branching hyphae |
| Sporulation | Spore production |
| Temperature range | 15-40℃ |
| Optimum temperature | 30℃ |
| pH range; Optimum | 5-10;6-8 |
| Habitat | soil |
| Oxygen requirement | aerobic |
| Biotic relationship | Free-living |
| Pathogenicity | Non-pathogen |
| Geographic location | Liuyang city, Hunan province, China |
| Sample collection | 2016 |
| Longitude/Latitude | 28°01’N,113°34’E |

**S2 Table**

S2 Table Project information of *Streptomyces rochei* FE-3-1

| Property | Term |
| --- | --- |
| Finishing quality | High-quality draft |
| Libraries used | Illumina Paired-End library |
| Sequencing platforms | Illumina Hiseq×10 + Pacbio |
| Fold coverage | 100× |
| Assemblers | SOAPdenovo v1.05 |
| Gene calling method | GeneMarkS+ |
| Project relevance | Bioremediation |

**S3 Table**

S3 Table The analysis of genomic islands

| Location | Island ID | Sample Name | Island Start | Island End | Length (bp) | Method | CDS No. |
| --- | --- | --- | --- | --- | --- | --- | --- |
| Chromosome | GI01 | FE_3_1 | 106730 | 112626 | 5896 | IslandPath-DIMOB | 7 |
| Chromosome | GI02 | FE_3_1 | 2437340 | 2446828 | 9488 | IslandPath-DIMOB | 9 |
| Chromosome | GI03 | FE_3_1 | 252362 | 260614 | 8252 | IslandPath-DIMOB | 10 |
| Chromosome | GI04 | FE_3_1 | 3752482 | 3762695 | 10213 | IslandPath-DIMOB | 10 |
| Chromosome | GI05 | FE_3_1 | 4307748 | 4315489 | 7741 | IslandPath-DIMOB | 7 |
| Chromosome | GI06 | FE_3_1 | 4500390 | 4524451 | 24061 | IslandPath-DIMOB | 22 |
| Chromosome | GI07 | FE_3_1 | 5424140 | 5438293 | 14153 | IslandPath-DIMOB | 9 |
| Chromosome | GI08 | FE_3_1 | 5570752 | 5583835 | 13083 | IslandPath-DIMOB | 12 |
| Chromosome | GI09 | FE_3_1 | 62477 | 95975 | 33498 | IslandPath-DIMOB | 34 |
| Chromosome | GI10 | FE_3_1 | 6570498 | 6577966 | 7468 | IslandPath-DIMOB | 7 |
| Chromosome | GI11 | FE_3_1 | 6645473 | 6729449 | 83976 | IslandPath-DIMOB | 60 |
| Chromosome | GI12 | FE_3_1 | 6747079 | 6785443 | 38364 | IslandPath-DIMOB | 47 |
| Chromosome | GI13 | FE_3_1 | 8150360 | 8170021 | 19661 | IslandPath-DIMOB | 21 |
| Chromosome | GI14 | FE_3_1 | 8216516 | 8239249 | 22733 | IslandPath-DIMOB | 23 |

**S4 Table**

**S4 Table The analysis of average nucleotide identity (ANI) between different strains**

| Sample Name | GCF_000827005.1 | GCF_003074055.1 | FE_3_1 | GCF_002155895.1 | GCF_000739045.1 | GCF_001905385.1 | GCF_003330845.1 | GCF_002154585.1 | GCF_003665435.1 |
| --- | --- | --- | --- | --- | --- | --- | --- | --- | --- |
| GCF_000827005.1 | 1 | 0.85286 | 0.854525 | 0.854533 | 0.853954 | 0.853036 | 0.852537 | 0.854052 | 0.853686 |
| GCF_003074055.1 | 0.85286 | 1 | 0.86538 | 0.865399 | 0.863957 | 0.863962 | 0.866947 | 0.866202 | 0.864542 |
| FE_3_1 | 0.854525 | 0.86538 | 1 | 0.990499 | 0.918671 | 0.92156 | 0.869267 | 0.868439 | 0.869793 |
| GCF_002155895.1 | 0.854533 | 0.865399 | 0.990499 | 1 | 0.91927 | 0.922082 | 0.869453 | 0.869151 | 0.869695 |
| GCF_000739045.1 | 0.853954 | 0.863957 | 0.918671 | 0.91927 | 1 | 0.924891 | 0.869005 | 0.867125 | 0.86876 |
| GCF_001905385.1 | 0.853036 | 0.863962 | 0.92156 | 0.922082 | 0.924891 | 1 | 0.868506 | 0.867379 | 0.868456 |
| GCF_003330845.1 | 0.852537 | 0.866947 | 0.869267 | 0.869453 | 0.869005 | 0.868506 | 1 | 0.868731 | 0.870536 |
| GCF_002154585.1 | 0.854052 | 0.866202 | 0.868439 | 0.869151 | 0.867125 | 0.867379 | 0.868731 | 1 | 0.883171 |
| GCF_003665435.1 | 0.853686 | 0.864542 | 0.869793 | 0.869695 | 0.86876 | 0.868456 | 0.870536 | 0.883171 | 1 |

**S5 Table**

**S5 Table The analysis of average amino acid identity (AAI) between different strains**

| Sample Name | GCF_000827005.1 | GCF_003074055.1 | FE_3_1 | GCF_002155895.1 | GCF_000739045.1 | GCF_001905385.1 | GCF_003330845.1 | GCF_002154585.1 | GCF_003665435.1 |
| --- | --- | --- | --- | --- | --- | --- | --- | --- | --- |
| GCF_000827005.1 | 100 | 74.07 | 73.78 | 74.09 | 74.04 | 73.72 | 74.11 | 73.99 | 74.28 |
| GCF_003074055.1 | 74.07 | 100 | 80.86 | 81.12 | 80.85 | 80.6 | 81.98 | 80.84 | 80.78 |
| FE_3_1 | 73.78 | 80.86 | 100 | 99.17 | 91.26 | 91.67 | 82.22 | 81.31 | 82.08 |
| GCF_002155895.1 | 74.09 | 81.12 | 99.17 | 100 | 91.31 | 91.88 | 82.31 | 81.25 | 82.29 |
| GCF_000739045.1 | 74.04 | 80.85 | 91.26 | 91.31 | 100 | 91.99 | 82.29 | 80.99 | 81.99 |
| GCF_001905385.1 | 73.72 | 80.6 | 91.67 | 91.88 | 91.99 | 100 | 81.75 | 81.12 | 81.81 |
| GCF_003330845.1 | 74.11 | 81.98 | 82.22 | 82.31 | 82.29 | 81.75 | 100 | 81.75 | 82.85 |
| GCF_002154585.1 | 73.99 | 80.84 | 81.31 | 81.25 | 80.99 | 81.12 | 81.75 | 100 | 85.68 |
| GCF_003665435.1 | 74.28 | 80.78 | 82.08 | 82.29 | 81.99 | 81.81 | 82.85 | 85.68 | 100 |

**S1 Fig.**


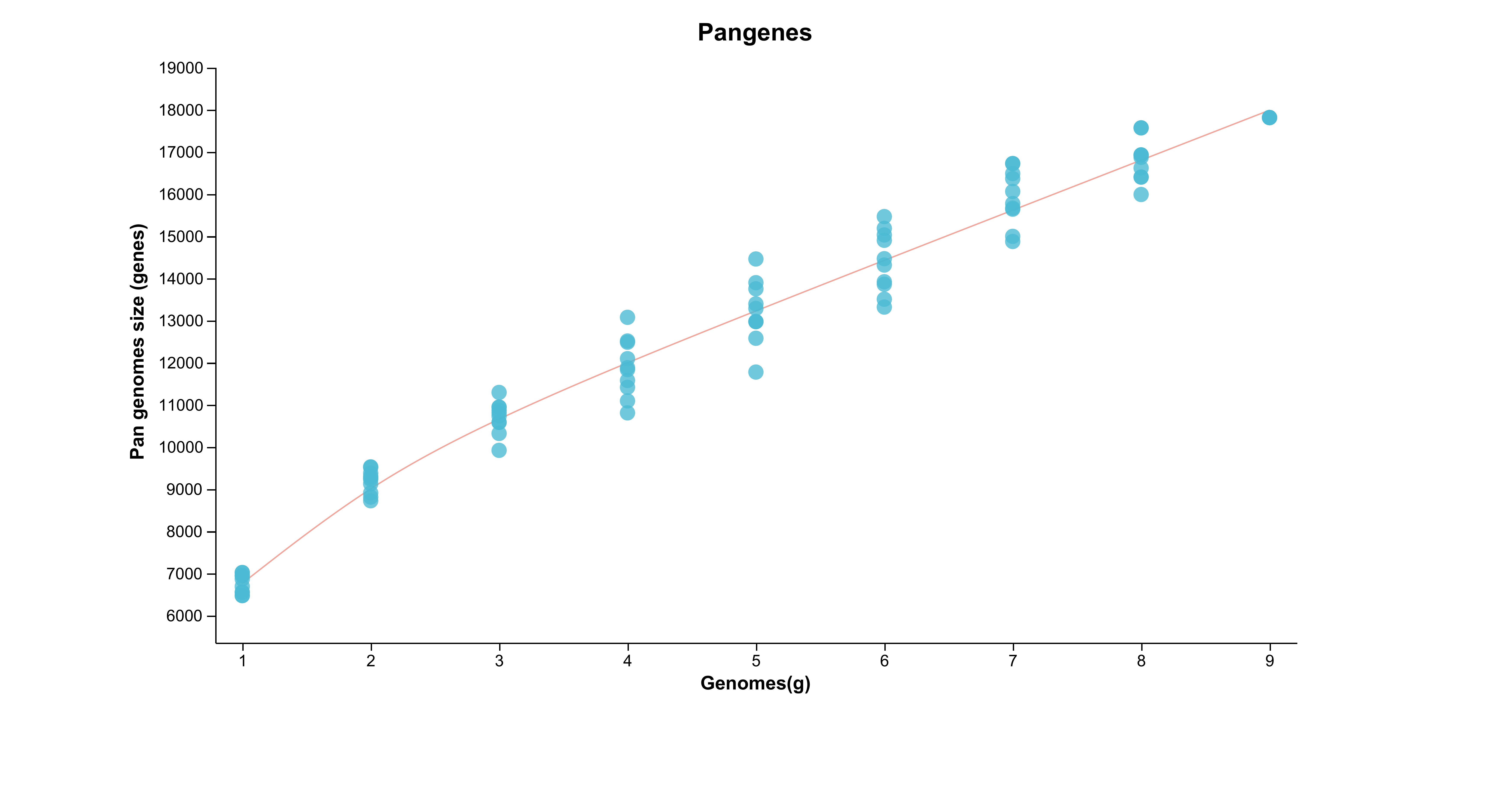


S1 Fig. Graph of the size of the pan-genome as a function of the number of genomes

**S2 Fig.**


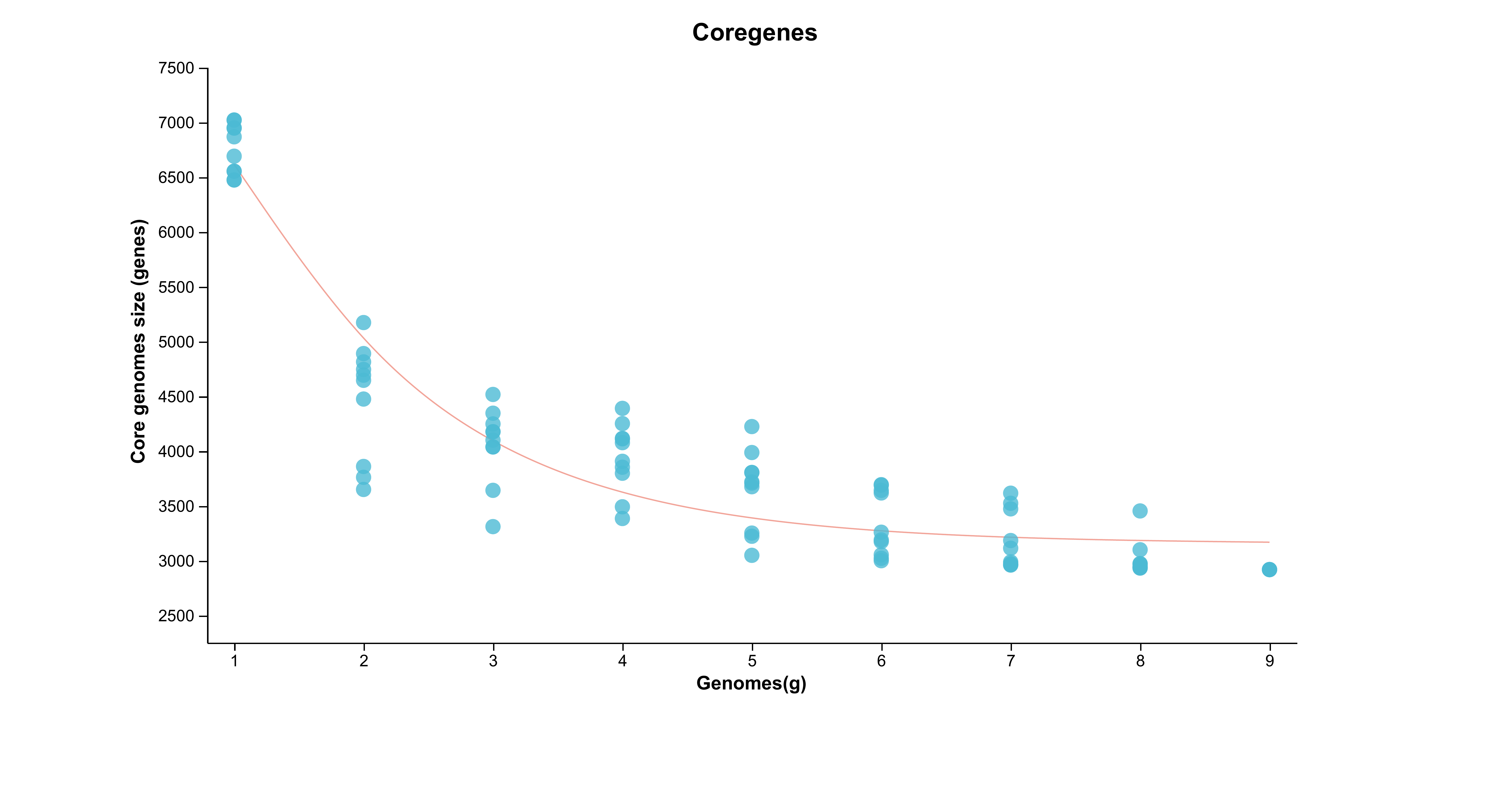


S2 Fig. Graph of the size of the core genes as a function of the number of genomes
