# Supplementary material for: Isolation, identification, and whole-genome sequencing of Streptomyces rochei FE-3-1 against Pyricularia oryzae
Source: PLoS One. 2025 Oct 29;20(10):e0331386. doi: 10.1371/journal.pone.0331386 (PMC12571248; doi:10.1371/journal.pone.0331386)
Supplement: Attachment — Submitted filename: Response to Reviewers.doc [file pone.0331386.s003.doc]

Dear editors,

Thank you very much for your E-mail with regard to our manuscript (PONE-D-24-32365) together with the comments from the reviewers. The comments from the reviewers were very helpful. We have responded each comment from the reviewers in a separate letter and taken all of these comments into account in preparing the revised manuscript. All of updates in the manuscript were marked via changing the font colour of the text. We believe that manuscript has been revised satisfactorily and hope it will be accepted for publication in PLOS ONE.

We thank again the reviewers for the helpful comments. Should you require any further information, please do not hesitate to ask.

Thanks very much for your attention and consideration.

Sincerely yours

Shiping Shan

**Responses to reviewers’ comments**

(Q refers to the question and R for the reply)

**To the Comments of Reviewer-1**

Q-1: Lines 87-99: You mention the potential use of *Streptomyces rochei* FE-3-1 as a biopesticide or biofertilizer. Could you expand on the next steps in product development or field trials? This would help position the findings for practical, real-world applications.

R-1:

1. Biological control product development steps

Rice is a staple food source for more than three billion people worldwide. However, rice is vulnerable to diseases, the most destructive among them being rice blast, which is caused by the fungus *Pyricularia oryzae*. This fungus attacks rice plants at all stages of development, causing annual losses of approximately 10-30% in various rice producing regions. Synthetic fungicides are often able to effectively control plant diseases, but some fungicides result in serious environmental and health problems. Therefore, there is growing interest in discovering and developing new, improved fungicides based on natural products as well as introducing alternative measures such as biocontrol agents to manage plant diseases.

*Streptomyces* are significant producers of antimicrobial secondary metabolites, which is not surprising given their ability to produce various bioactive compounds. The members of *Streptomyces* are well-known for their ability to produce a variety of bioactive compounds with different bioactivities such as antibacterial, antifungal, anticancer, and antioxidant properties. These bioactive compounds have important applications in various fields. For example, approximately 75% of commercially useful antibiotics were derived from the genus *Streptomyces* and they are thus the primary antibiotic-producing organisms exploited by the pharmaceutical industry.

Regarding the strain in product development, we are prepared to undertake the following actions.

(1) In vitro experiments ( Completed)

In this study, *Streptomyces rochei* FE-3-1 was isolated from rice rhizosphere soil and exhibited inhibitory effect on plant pathogen fungus *Pyricularia oryzae*, indicating its potential as a source of anti-*Pyricularia oryzae* compound. The results demonstrated that the colony radius of pathogenic fungi *Pyricularia oryzae* on the plate inoculated with antagonistic strain FE-3-1 was 8.00±0.035 mm, whereas that of control pathogen fungi was 30.00±0.046 mm, resulting in an inhibition rate of 73.33±0.079 %. This suggests that *Streptomyces rochei* FE-3-1 has greater inhibition against the rice blast fungus.

(2) Greenhouse (in progress)

The limitation of laboratory experiments is that they only prove the antagonistic activity of *Streptomyces rochei* FE-3-1 against *Pyricularia oryzae* under certain conditions. The antagonism exhibited by *Streptomyces rochei* FE-3-1 in laboratory experiments might not necessarily reflect antagonism under greenhouse or field experiments. Greenhouse are conducted for the purpose of further evaluating the efficacy of *Streptomyces rochei* FE-3-1 as biocontrol agents. Currently, this work is underway.

(3) Bioactive compounds with antifungal activity (not yet done)

The suppression of rice blast in the greenhouse by certain *Streptomyces* might indicate the presence of bioactive compound(s) with antifungal activity against *Pyricularia oryzae* in the culture filtrates. Some well-known antibiotics produced by *Streptomyces* have been used as fungicides for the control of rice blast, for instance, Blasticidin-S and Kasugamycin. Therefore, bioactive compounds derived from *Streptomyces* can be used for the management of fungal plant diseases as an alternative to synthetic fungicides. These natural compounds are biologically synthesized and often biodegradable. Thus, they may be useful for the development of agricultural fungicides that are more pathogen-specific with minimal sideeffects toward the environment.

1. Biofertilizer product development steps

Some *Streptomyces* species are plant growth-promoting *Streptomycetes* that have established favorable interactions with plants. Generally, plant growth-promoting *streptomycetes* promote plant growth via a variety of mechanisms, including nitrogen fixation, phytohormone generation, 1-aminocyclopropane-1-carboxylic acid (ACC) deaminase, and siderophore production, thereby alleviating abiotic or biotic stress to plants. *Streptomyces* spp. were also reported to promote their colonization in plants by producing siderophores, as well as assist in obtaining iron nutrition and inhibit the growth of plant pathogens through iron competition.

Regarding the strain in product development, we are prepared to undertake the following actions.

(1) Genes related to plant growth promotion traits (Completed)

According to Prokka annotations, the genome of *Streptomyces rochei* FE-3-1 harbors numerous genes associated with promoting plant growth, including those involved in phosphate solubilization, potassium solubilization, nitrogen assimilation and siderophore production (Table 1). Additionally, the presence of genes responsible for chitinase production and related lyases, such as protease and lipase, suggests potent antifungal properties, and antifungal activity of the strain has been confirmed. Furthermore, the peroxidase/catalase-related genes were also detected within the genome of *Streptomyces rochei* FE-3-1 and the production of peroxidase and catalase by strain FE-3-1 may be an important mechanism of plant resistance to oxidative stress.

Table 1 Some genes related to the plant growth promotion within *Streptomyces rochei* FE-3-1 according to Prokka annotations

| Traits | Genes | Products |
| --- | --- | --- |
| Nitrogen assimilation | *nir*B, *nir*D | Nitrite reductase |
|  | *nar*K, | Nitrate/nitrite transporter |
|  | *nar*I, *nar*J, *nar*H, *nar*G | Nitrate reductase |
| Phosphate solubilization | *rsb*U_P, *prp*C | Protein phosphatase |
|  | *pho*D | Alkaline phosphatase D |
|  | *rhn*A-*cob*C | RNase H/acid phosphatase |
|  | *maf* | Nucleotide pyrophosphatase |
|  | *ppa* | Inorganic diphosphatase |
|  | *gpm*B | Histidine phosphatase |
| Potassium solubilization | *kdp*A, *kdp*B, *kdp*C | Potassium-transporting ATPase |
|  | *trk*A | Potassium uptake protein |
|  | *cvr*A | Potassium/proton antiporter |
| Iron sequestration | *fhu*D | Iron-siderophore ABC transporter substrate-binding protein |
|  | - | Siderophore-interacting protein |
| Phytohormone synthesis | *trp*C | Indole-3-glycerol phosphate synthase |
| Biocatalyst | *snp*A | Zinc-dependent metalloprotease |
|  | *fts*H, *htp*X | Zinc metalloprotease |
|  | *hya*D | Hydrogenase maturation protease |
|  | - | Lipase |
|  | *plc* | Phospholipase |
|  | *pnb*A | Carboxylesterase/lipase |
|  | - | Chitinase |
|  | *amy*A | Alpha-amylase |
|  | *kat*E | Catalase |
|  | *kat*G | Catalase/peroxidase |

(2) Bioassays (in progress)

The bioassays are being performed to reveal multiple plant growth-promoting properties of *Streptomyces rochei* FE-3-1, including phosphate solubilization, potassium solubilization, nitrogen assimilation, siderophore production and indole-3-acetic acid (IAA).

(3) Preparation of spore suspension (completed)

Mature *Streptomyces* spores were selected using an inoculation ring and uniformly dispersed in sterile distilled water to prepare spore suspension. The suspension was counted with a blood cell counting plate and diluted to the requisite application concentration.

(4) Pot experiment (not yet done)

To evaluate the plant growth promotion activity of *Streptomyces rochei* FE-3-1 on rice plants, a pot or field experiments will be performed. rice plants were inoculated with 30 mL of different concentrations of *Streptomyces rochei* FE-3-1 spore suspension (10^7^, 10^8^, 10^9^ CFU/mL) with root drenching methods for each treatment. Rice plants treated with 30 mL sterile distilled water were used as a control. Biometric properties (dry weight, fresh weight, root length, shoot length and rice yield) were measured and statistically analyzed at the same time. Three independent experiments were conducted, and each treatment contained five replicate pots, with each replicate pot containing 3 rice plants.

Q-2: Lines 111-121: Please provide more details on the statistical methods used to validate the inhibition rates observed in the antagonistic activity assay against *Pyricularia oryzae*. Including specific statistical tests will add rigor to the data and help readers assess the reliability of the results. Additionally, have you assessed the ability of *Streptomyces rochei* FE-3-1 to control *Pyricularia oryzae*-caused disease in actual rice plants, beyond the in vitro antagonistic assays? If so, please provide detailed information on these trials, as it would greatly enhance the practical relevance of your findings.

R-2: Each treatment was replicated three times, and the colony diameter was determined by the cross-crossing method using vernier calipers. Means and standard errors of the data were calculated using Excel 2010 (Microsoft Corporation, USA). All data collected were statistically analyzed according to Duncan’s multiple range test (P=0.05).

The above contents have been added on Page 6 Line 119 and Page 8 Line 165-168, and the changes have been highlighted in red.

The statistical results are as follows:

The results demonstrated that the colony radius of pathogenic fungi *Pyricularia oryzae* on the plate inoculated with antagonistic strain FE-3-1 was 8.00±0.035mm, whereas that of control pathogen fungi was 30.00±0.046 mm, resulting in an inhibition rate of 73.33±0.079%.

Table 1 Data statistics table of inhibition rate

| Colony radius and inhibition rate | Repeat 1 | Repeat 2 | Repeat 3 | Mean± standard deviation |
| --- | --- | --- | --- | --- |
| The colony radius of pathogenic fungi with strain FE-3-1 (mm) | 7.98 | 7.98 | 8.04 | 8.00±0.035 |
| The colony radius of pathogenic fungi (mm) | 29.96 | 29.99 | 30.05 | 30.00±0.046 |
| The inhibition rate (%) | 73.36 | 73.39 | 73.24 | 73.33±0.079 |

The above contents have been added on Page 10 Line 190-194, and the changes have been highlighted in red.

The limitation of laboratory experiments is that they only prove the antagonistic activity of *Streptomyces rochei* FE-3-1 against *Pyricularia oryzae* under certain conditions. The antagonism exhibited by *Streptomyces* in laboratory experiments might not necessarily reflect antagonism under greenhouse. Greenhouse or growth chamber experiments are conducted for the purpose of further evaluating the efficacy of *Streptomyces rochei* FE-3-1 as biocontrol agents. Currently, this work is underway. These trials would greatly enhance the practical relevance of our findings.

Q-3: Lines 229-237: The identification of heavy metal resistance genes is intriguing, but it would be beneficial to discuss how these genes enhance the strain’s survival and effectiveness in agricultural environments that are subject to pollution or heavy metal contamination.

R-3: The soil heavy metal pollution is an important limiting factor for the survival of the strain in the environment. Bacterial mechanisms to combat contamination of the environment by toxic metals are of great environmental and economic significance. Bacteria have developed several mechanisms for resisting and remediating heavy metals for their survival. Certain bacterial metabolic mechanisms exist that are specifically dedicated towards immobilizing and reducing bioavailability of heavy metals. The immobilization can be achieved by sorption to cell components and exopolymers, transport and intracellular sequestration or precipitation of metal ions as insoluble organic and inorganic compounds. Some of the other processes used by bacteria to combat heavy metal toxicity include membrane filtration, ion exchange, adsorption, coagulation, oxidation-reduction, electrochemical treatment, evaporation, and chemical precipitation. All these transformations that alters the mobility and toxicity of heavy metals are of immense biogeochemical significance and hold bioremediation potential.

The strain FE-3-1 harbors multiple putative functional proteins associated with different heavy metals (Cadmium, Arsenic and Copper) or metal (Zinc), and putative functions including transporters, P-type ATPase and resistance proteins, and so on, and they provide resistance to copper, zinc, cadmium, and arsenic. These heavy metal resistances enhance the ability of the strain FE-3-1 to survive in extreme environments, as well as to provide a foundation for its colonization of the rhizosphere and promotion of plant growth.

[1] Pal A, Bhattacharjee S, Saha J, Sarkar M, Mandal P. Bacterial survival strategies and responses under heavy metal stress: a comprehensive overview. Crit Rev Microbiol. 2022;48(3):327-355. <https://doi.org/10.1080/1040841X.2021.1970512.> PMID: 34473592.

The above contents have been added on Page 26-27 Line 467-479. The reference is cited on Page 40 Line 757-760, and the changes have been highlighted in red.

Q-4: Lines 240-253: Could you clarify how the genes related to plant growth promotion, such as those for phosphorus and potassium solubilization, enhance the biocontrol efficiency of *Streptomyces rochei* FE-3-1? Understanding the synergy between plant growth and pathogen inhibition would be valuable.

R-4: According to Prokka annotations, the genome of *Streptomyces rochei* FE-3-1 harbors numerous genes associated with promoting plant growth, including those involved in phosphate solubilization, potassium solubilization, nitrogen assimilation and siderophore production.

Nitrogen, one of the most vital elements, plays an important role in plant growth and various metabolic activities. Conversion of nitrogen to ammonia (NH_3_) by *Streptomyces rochei* FE-3-1 using a nitrogenase protein complex. Phosphorus (P) is the second most vital macroelement after nitrogen and is required for adequate plant nutrition. It has key roles in the processes of photosynthesis, energy transfer, and various plant metabolic processes. In most soils, P is present in excess, but not in a form available to plants. only 0.1% of total soil phosphorus is available for plant use. Plant growth-promoting microbes offer a suitable option to enhance crop productivity. These microbes increase phosphorus availability in soil by mediating the mineralization of organic P in soil. Another vital macronutrient after nitrogen and phosphorus is potassium (K), which is the seventh most abundant element in the Earth’s crust. It plays a key role in various metabolic, growth, and development-related processes in plants. Moreover, potassium is responsible for activating more than 80 enzymes involved in photosynthesis activity, nitrate reduction, starch synthesis, and various energy metabolic processes. *Streptomyces rochei* FE-3-1 may function as biocontrol agents via a variety of mechanisms. An increase in plant vigor against various biotic stresses, diseases, and pests is also regulated by an adequate supply of N, P and K.

The above contents have been added on Page 24 Line 429-433, and the changes have been highlighted in red.

The siderophore synthesis is one of the most important mechanism for preventing phytopathogen propagation. These siderophores serve as iron chelators, binding the majority of the iron in the rhizosphere. As a result, the lack of iron in the rhizosphere inhibits bacterial and fungal pathogen proliferation. In addition, the development of secondary metabolites may be a direct result of plant growth.

Q-5: Lines 257-274: The manuscript identifies several biosynthetic gene clusters, such as those for candicidin and streptothricin, which are related to antimicrobial activity. Expanding on how these clusters contribute to the biocontrol properties of *Streptomyces rochei* FE-3-1 would strengthen the link between the genomic data and the biological functions of the strain.

R-5: A total of 31 biosynthetic gene clusters were identified within the genome of *Streptomyces rochei* FE-3-1 via antiSMASH. Four of these biosynthetic gene clusters exhibited high similarity to known metabolites with antimicrobial properties such as candicidin, streptothricin, lipopeptide, and albaflavenone.

The candicidin complex was initially isolated from Streptomyces griseus IMRU3570. It demonstrates a potent inhibitory activity against fungi. The candicidin complex was regarded as the prototype of aromatic polyene macrolides. Similar to other polyketide polyenes, candicidin can interact with ergosterol existing in the membranes of fungi to form a transmembrane channel, resulting in K+ leakage and causing cell death.

Streptothricin is an N-glucoside antibiotic, which exists in different *Streptomyces* and is one of the earliest antibiotics isolated from *Streptomyces* and has broad-spectrum activity against Gram-positive, Gram-negative bacteria and pathogenic fungi. It inhibits protein biosynthesis in prokaryotic cells by inhibiting polypeptide synthesis via ribosome interactions.

Lipopeptide antibiotics represent the most recent addition to the antibiotic family, with molecular weights typically ranging from 1000-1600 Da. They are synthesized via a non-ribosomal multi-enzyme biosynthesis pathway. Surfactin, iturin, and fengycin are exemplary lipid peptides known for their intricate effects on plant pathogenic fungi.

The tricyclic sesquiterpene albaflavenone was first isolated from *Streptomyces albidoflavus*, and is known to exhibit antibacterial properties. The pathway for the biosynthesis of albaflavenone was recently elucidated in S. coelicolor A3(2).

The above contents have been added on Page 27-28 Line 491-503, and the changes have been highlighted in red.

[1] Ji Z, Wang M, Zhang J, Wei S, Wu W. Two new members of streptothricin class antibiotics from Streptomyces qinlingensis sp. nov. J Antibiot (Tokyo). 2007; 60:739-744. <https://doi.org/10.1038/ja.2007.96.> PMID: 18276997

[2] Gil JA, Campelo-Diez AB. Candicidin biosynthesis in Streptomyces griseus. Appl. Microbiol. Biotechnol. 2003; 60: 633-642. <https://doi.org/10.1007/s00253-002-1163-9.> PMID: 12664141

[3] Zhao B, Lin X, Lei L, Lamb DC, Kelly SL, Waterman MR, et al. Biosynthesis of the sesquiterpene antibiotic albaflavenone in Streptomyces coelicolor A3(2). J. Biol. Chem. 2008; 283: 8183-8189. <https://doi.org/10.1074/jbc.M710421200.> PMID: 18234666

[4] Stein T. Bacillus subtilis antibiotics: structures, syntheses and specific functions. Mol. Microbiol. 2005; 56(4):845-857. <https://doi.org/10.1111/j.1365-2958.2005.04587.x.> PMID: 15853875

[5] Kim PI, Ryu J, Kim YH, Chi YT. Production of biosurfactant lipopeptides Iturin A, fengycin and surfactin A from Bacillus subtilis CMB32 for control of Colletotrichum gloeosporioides. J Microbiol Biotechnol. 2010; 20(1):138-145. <https://doi.org/10.4014/jmb.0905.05007.> PMID: 20134245

[6] Lam VB, Meyer T, Arias AA, Ongena M, Oni FE, Höfte M. Bacillus cyclic lipopeptides Iturin and Fengycin control rice blast caused by Pyricularia oryzae in potting and acid sulfate soils by direct antagonism and induced systemic resistance. Microorganisms. 2021; 9(7):1441. <https://doi.org/10.3390/microorganisms9071441.> PMID: 34361878

[7] Liao JH, Chen PY, Yang YL, Kan SC, Hsieh FC, Liu YC. Clarification of the antagonistic effect of the lipopeptides produced by Bacillus amyloliquefaciens BPD1 against Pyricularia oryzae via in situ MALDI-TOF IMS analysis. Molecules. 2016; 21(12):1670. <https://doi.org/10.3390/molecules21121670.> PMID: 27918491

[8] Omoboye OO, Geudens N, Duban M, Chevalier M, Flahaut C, Martins JC, et al. Pseudomonas sp. COW3 produces new bananamide-type cyclic lipopeptides with antimicrobial activity against Pythium myriotylum and Pyricularia oryzae. Molecules. 2019; 24(22):4170. <https://doi.org/10.3390/molecules24224170.> PMID: 31744250

Q-6: Lines 257-277: It would be helpful to include a comparison of the identified secondary metabolites with known commercial biocontrol agents in terms of their effectiveness and range of activity. This could give a clearer perspective on the potential competitive advantage of *Streptomyces rochei* FE-3-1.

R-6: Based on different *Streptomyces* strains, many commercial biocontrol agents, such as Actinovate (*Streptomyces lydicus* WYEC108), Rhizovit (*Streptomyces rimosus*), and Mycostop (*Streptomyces griseoviridis* K61), have been developed for agricultural purposes, and these fungicides show resistance to a variety of pathogens (S6 Table). Therefore, *Streptomyces* are important resources for agricultural fungicides or biofertilizers. In this study, a total of 31 biosynthetic gene clusters were identified within the genome of *Streptomyces rochei* FE-3-1, its metabolites exhibited potent antagonistic activity against plant pathogen *Pyricularia oryzae* by the agar disk diffusion method. Four of these biosynthetic gene clusters exhibited high similarity to known metabolites with antifungal and antibacterial properties such as candicidin, streptothricin, lipopeptide, and albaflavenone. Some gene clusters were also related to active metabolites such as isorenieratene, desferrioxamine, hopene, ectoine, sapB, fluostatins, 7-prenylisatin, melanin, borrelidin and geosmin. Therefore, this strain is predicted to have inhibitory activities against a variety of pathogens, and we will further explore these active metabolites in the future.

S6 Table. Commercialized streptomyces based biopesticides

| Biocontrol agent(s) | Active ingredient | Target pathogen(s) | Biocontrol  mechanism | Country |
| --- | --- | --- | --- | --- |
| Actinovate | s*treptomyces lydicus* WYEC108 | *Fusarium* spp., *Rhizoctonia* spp.,  *Pythium* spp., *Phytophthora*  spp., *Erisiphe* spp., *Sphaeroteca*  spp., *Laveillula* spp., *Sclerotinia*  spp. | Antibiosis and  hyperparasitism | European Union |
| Rhizovit | *Streptomyces rimosus* | *Pythium* spp., *Fusarium* spp.,  *Phomopsis* spp., *Phytophthora*  spp., *R. solani*, *A. brassicola*,  *Botrytis* spp., *Fusarium* spp. | Antibiosis | - |
| Mycostop | *Streptomyces griseoviridis* K61 | *Ceratocystis radicicola*,  *Alternaria* spp., *Rhizoctonia*  *solani*, *Fusarium* spp.,  *Phytophthora* spp., *Pythium* spp. | Competition,  hyperparasitism, and  antibiosis | Canada |

[1] Marine SC, Newark MJ, Korir RC, Everts KL. Evaluation of Rotational Biopesticide Programs for Disease Management in Organic Cucurbit Production. Plant Dis. 2016;100(11):2226-2233. <https://doi.org/10.1094/PDIS-02-16-0252-RE.> PMID: 30682910

[2] Himmelstein JC, Maul JE, Everts KL. Impact of Five Cover Crop Green Manures and Actinovate on *Fusarium Wilt* of Watermelon. Plant Dis. 2014 Jul;98(7):965-972. <https://doi.org/10.1094/PDIS-06-13-0585-RE.> PMID: 30708841

[3] Minuto A, Spadaro D, Garibaldi A, Gullino M L. Control of soilborne pathogens of tomato using a commercial formulation of *Streptomyces griseoviridis* and solarization. Crop Protection, 2006, 25: 468-475. https://doi.org/10.1016/j.cropro.2005.08.001

[4] Silva GC, Kitano IT, Ribeiro IAF and Lacava PT. The Potential Use of

*Actinomycetes* as Microbial Inoculants and Biopesticides in Agriculture. Front. Soil Sci. 2022; 2:833181. https://doi.org/10.3389/fsoil.2022.833181.

The above contents have been added on Page 28-29 Line 516-525, The reference is cited on Page 36 Line 673-676, and S6 Table has been inserted into the supplementary materials on Page 31 Line 560, and the changes have been highlighted in red.

Q-7: Lines 333-362: The manuscript mentions unique homologous genes found in *Streptomyces rochei* FE-3-1. It would be beneficial to discuss how these genes might contribute to the strain’s unique abilities or adaptations, especially in comparison with other *Streptomyces* species.

R-7: In this study, comparative genomic analysis of nine distinct Streptomyces strains demonstrated that 244 unique gene clusters were specific to the strain FE-3-1. Among these unique homologous genes, such as nitrite reductase [EC:1.7.2.1], pyruvate carboxylase [EC:6.4.1.1], and polyphosphate kinase [EC:2.7.4.34], etc. The enzyme proteins encoded by these gene clusters play crucial roles in promoting growth, synthesizing secondary metabolites and tolerating external environmental stress. Moreover, these unique homologous genes may contribute to the strain's distinctive capabilities.

The above contents have been added on Page 23 Line 402-410, and the changes have been highlighted in red.

Q-8: Lines 364-374: Finally, your study contributes significantly to microbial genome mining. Could you emphasize how your findings advance the field of genome-guided discovery of biocontrol agents and antibiotics?

R-8: The advent of the genomic era has opened up enormous possibilities for the discovery of new natural products. Microbial genome mining is an alternative approach to more traditional methods for the discovery of novel secondary metabolites, which continue to serve as scaffolds for further embellishment by combinatorial biosynthesis for the development of products for crop protection, and numerous biotechnological applications.

Many microbial metabolites remain undiscovered using traditional low-throughput methods due to the association of natural products with cryptic genes, which often require external stimulation for microbes to produce. Genome mining facilitates the identification of those cryptic, uncharacterized biosynthetic gene clusters responsible for synthesizing natural products, which are likely novel.

Among microorganisms, bacteria, because of their relatively simple and small genome sizes, have been the most studied for genome mining. *Streptomyces* spp., known for prolific secondary metabolite production, was found to encode even more secondary metabolites through genome mining. It has been estimated that ~ 10^26^ *Actinomycete* colony-forming units exist in the top 10 cm of soil covering the Earth, but only ~ 10^7^ *Actinomycetes* have been screened for secondary metabolite production over the past fifty years. Using whole-genome sequencing and bioinformatics, genome mining has unveiled many unknown metabolites from diverse bacteria.

This study provided comprehensive insights into the genomic features and functional elements present in *Streptomyces rochei* FE-3-1. The strain FE-3-1 possesses a rich repertoire of biosynthetic gene clusters with the potential to produce diverse and novel natural products. The results show the capability of this bacterium to produce a diverse and large number of natural products that may have potential antibiotic or antimicrobial activity against agricultural crop fungal pathogens, making this bacterium an excellent microbial candidate for mining novel natural products. Decoding microbial genomes and harnessing their natural products can address critical challenges in agriculture.

[1] Kenshole E, Herisse M, Michael M, Pidot SJ. Natural product discovery through microbial genome mining. Curr Opin Chem Biol. 2021;60:47-54. https://doi.org/10.1016/j.cbpa.2020.07.010. PMID: 32853968
[2] Bachmann BO, Van Lanen SG, Baltz RH. Microbial genome mining for accelerated natural products discovery: is a renaissance in the making? J Ind Microbiol Biotechnol. 2014;41(2):175-84. <https://doi.org/10.1007/s10295-013-1389-9.> PMID: 24342967
[3] Tamang P, Upadhaya A, Paudel P, Meepagala K, Cantrell CL. Mining Biosynthetic Gene Clusters of *Pseudomonas vancouverensis* Utilizing Whole Genome Sequencing. Microorganisms. 2024; 12(3): 548. <https://doi.org/10.3390/microorganisms12030548.> PMID: 38543599.

The above contents have been added on Page 22 Line 376-382, the reference is cited on Page 37 Line 696-699, and the changes have been highlighted in red.

Q-9: Introducing biocontrol agents into agricultural systems carries some risk. Have you considered any potential environmental impacts or risks associated with using *Streptomyces rochei* FE-3-1? If so, how might these be mitigated?

R-9: In this study, *Streptomyces rochei* FE-3-1was isolated from the rhizosphere soil of rice plants. The strain was an indigenous rhizosphere microorganism of rice and was reintroduced into the rhizosphere soil after propagation, presenting a low environmental risk. Under laboratory conditions, the strain has undergone hemolysis tests on blood plates, and the results are negative (Figure 1). This also indicates that the risk to the surrounding environment is low. In the subsequent process of product development, animal experiments should also be conducted strictly to guarantee the safety of the strain to the surrounding environment.

1

2

3

Figure 1 Hemolysis experiment of the strain FE-3-1

1, 2, 3: The strain FE-3-1

Q-10: PLOS requires an ORCID iD for the corresponding author in Editorial Manager.

R-10: The ORCID iD has been registered online:

My ORCID iD: 0009-0004-5537-826X

My ORCID record is <https://orcid.org/0009-0004-5537-826X>

Q-11: When you resubmit, please ensure that you provide the correct grant numbers for the awards you received for your study in the ‘Funding Information’ section.

R-11: We have been checked grant numbers for the awards for this study in the ‘Funding Information’ section.

Q-12: Please ensure that your manuscript meets PLOS ONE's style requirements, including those for file naming.

R-12:

(1) Authors’ contributions has been revised to meet PLOS ONE's style requirements on Page 31 Line 564-573, and the changes have been highlighted in red.
(2) References have been revised meet PLOS ONE's style requirements on Page 32-42 Line 592-800, and the changes have been highlighted in red.
