# Supplementary material for: Isolation, identification, and whole-genome sequencing of Streptomyces rochei FE-3-1 against Pyricularia oryzae
Source: PLoS One. 2025 Oct 29;20(10):e0331386. doi: 10.1371/journal.pone.0331386 (PMC12571248; doi:10.1371/journal.pone.0331386)
Supplement: Attachment — Submitted filename: Response_to_Reviewers_auresp_2.doc [file pone.0331386.s004.doc]

Dear editors,

Thank you very much for your E-mail with regard to our manuscript (PONE-D-24-32365R1) together with the comments from the reviewers. The comments from the reviewers were very helpful. We have responded each comment from the reviewers in a separate letter and taken all of these comments into account in preparing the revised manuscript. All of updates in the manuscript were marked via changing the font colour of the text. We believe that manuscript has been revised satisfactorily and hope it will be accepted for publication in PLOS ONE.

We thank again the reviewers for the helpful comments. Should you require any further information, please do not hesitate to ask.

Thanks very much for your attention and consideration.

Sincerely yours

Shiping Shan

**Responses to reviewers’ comments**

(Q refers to the question and R for the reply)

**To the Comments of Reviewer-1**

Q-1: The author has made corrections according to the suggestions, but minor errors remain, such as the need to italicize the scientific name Streptomyces. It is therefore recommended that the author thoroughly check the manuscript for accuracy.

R-1: All the authors thoroughly reviewed the entire manuscript and revised all scientific names to be presented in italics on Page23 Line403, Page29 Line518, Page31 Line561, Page36 Line661-662, Page37 Line698. and the changes have been highlighted in red.
