# Supplementary material for: Isolation, identification, and whole-genome sequencing of Streptomyces rochei FE-3-1 against Pyricularia oryzae
Source: PLoS One. 2025 Oct 29;20(10):e0331386. doi: 10.1371/journal.pone.0331386 (PMC12571248; doi:10.1371/journal.pone.0331386)
Supplement: Attachment — Submitted filename: Response_to_Reviewers_auresp_3.doc [file pone.0331386.s005.doc]

Dear editors,

Thank you very much for your E-mail with regard to our manuscript (PONE-D-24-32365R2) together with the comments from the reviewers. The comments from the reviewers were very helpful. We have responded each comment from the reviewers in a separate letter and taken all of these comments into account in preparing the revised manuscript. All of updates in the manuscript were marked via changing the font colour of the text. We believe that manuscript has been revised satisfactorily and hope it will be accepted for publication in PLOS ONE.

We thank again the reviewers for the helpful comments. Should you require any further information, please do not hesitate to ask.

Thanks very much for your attention and consideration.

Sincerely yours

Shiping Shan

**Responses to reviewers’ comments**

(Q refers to the question and R for the reply)

**To the Comments of Reviewer**

Q-1:Lines 87-99: You mention the potential use of *Streptomyces rochei* FE-3-1 as a biopesticide or biofertilizer. Could you expand on the next steps in product development or field trials? This would help position the findings for practical, real-world applications.

R-1: (I) Biological Control Product Development Steps

Rice is a staple food source for over three billion people worldwide. However, rice is vulnerable to diseases, the most destructive being rice blast, caused by the fungus *Pyricularia oryzae*. This fungus attacks rice plants at all developmental stages, causing annual yield losses of approximately 10-30% in various rice-producing regions. While synthetic fungicides often effectively control plant diseases, some pose serious environmental and health risks. Consequently, there is growing interest in discovering and developing new, improved natural-product-based fungicides, as well as introducing alternative measures like biocontrol agents, for disease management.

*Streptomyces* species are significant producers of antimicrobial secondary metabolites, reflecting their capacity to synthesize diverse bioactive compounds. Members of this genus are well-known for producing compounds with antibacterial, antifungal, anticancer, and antioxidant properties, which have important applications across various fields. Notably, approximately 75% of commercially useful antibiotics originate from *Streptomyces*, making them primary antibiotic producers for the pharmaceutical industry.

Regarding the strain FE-3-1 in product development, we are undertaking the following actions:

1. In Vitro Experiments (Completed)

*Streptomyces rochei* strain FE-3-1 was isolated from rice rhizosphere soil and demonstrated inhibitory effects against the plant pathogenic fungus *Pyricularia oryzae*, indicating its potential as a source of anti-*Pyricularia oryzae* compounds. Results showed the colony radius of *P. oryzae* on plates inoculated with antagonistic strain FE-3-1 was 8.00±0.035 mm, compared to 30.00± 0.046 mm for the control, resulting in an inhibition rate of 73.33±0.079%. This suggests *Streptomyces rochei* FE-3-1 exhibits strong inhibition against the rice blast fungus under laboratory conditions.

2. Greenhouse Trials (In Progress)

A limitation of laboratory experiments is that they only demonstrate antagonistic activity under specific controlled conditions; activity observed in vitro may not necessarily translate to greenhouse or field environments. Greenhouse trials are currently underway to further evaluate the efficacy of *Streptomyces rochei* FE-3-1 as a biocontrol agent against rice blast under more realistic conditions.

3. Bioactive Compounds with Antifungal Activity (Not Yet Done)

Suppression of rice blast in greenhouse trials by certain *Streptomyces* strains may indicate the presence of bioactive antifungal compound(s) against *P. oryzae* in their culture filtrates.Well-known antibiotics produced by *Streptomyces*, such as Blasticidin-S and Kasugamycin, have been used as fungicides for rice blast control. Therefore, bioactive compounds derived from *Streptomyces* offer an alternative to synthetic fungicides for managing fungal plant diseases. These natural compounds are biologically synthesized and often biodegradable, potentially enabling the development of more pathogen-specific agricultural fungicides with minimal environmental side effects.

(II) Biofertilizer Product Development Steps

Some *Streptomyces* species are plant growth-promoting rhizobacteria (PGPR) that establish beneficial interactions with plants. Generally, plant growth-promoting *streptomycetes* enhance plant growth through various mechanisms, including nitrogen fixation, phytohormone production, 1-aminocyclopropane-1-carboxylic acid (ACC) deaminase activity, and siderophore production, thereby alleviating abiotic or biotic stresses. *Streptomyces* spp. can also promote plant colonization by producing siderophores, which aid in iron acquisition and inhibit plant pathogen growth through iron competition.

Regarding the strain FE-3-1 in product development, we are undertaking the following actions:

1. Genes Related to Plant Growth Promotion Traits (Completed)

Prokka genome annotation revealed that *Streptomyces rochei* FE-3-1 harbors numerous genes associated with plant growth promotion (Table 1). These include genes involved in solubilizing phosphate and potassium, nitrogen assimilation, and siderophore production. The presence of genes responsible for chitinase production and related lyases (e.g., protease, lipase) suggests potent antifungal properties, which have been experimentally confirmed. Furthermore, peroxidase/catalase-related genes were detected in the FE-3-1 genome; the production of these enzymes may represent an important mechanism for enhancing plant resistance to oxidative stress.

Table 1 Some genes related to the plant growth promotion within *Streptomyces rochei* FE-3-1 according to Prokka annotations

| Traits | Genes | Products |
| --- | --- | --- |
| Nitrogen assimilation | *nir*B, *nir*D | Nitrite reductase |
|  | *nar*K, | Nitrate/nitrite transporter |
|  | *nar*I, *nar*J, *nar*H, *nar*G | Nitrate reductase |
| Phosphate solubilization | *rsb*U_P, *prp*C | Protein phosphatase |
|  | *pho*D | Alkaline phosphatase D |
|  | *rhn*A-*cob*C | RNase H/acid phosphatase |
|  | *maf* | Nucleotide pyrophosphatase |
|  | *ppa* | Inorganic diphosphatase |
|  | *gpm*B | Histidine phosphatase |
| Potassium solubilization | *kdp*A, *kdp*B, *kdp*C | Potassium-transporting ATPase |
|  | *trk*A | Potassium uptake protein |
|  | *cvr*A | Potassium/proton antiporter |
| Iron sequestration | *fhu*D | Iron-siderophore ABC transporter substrate-binding protein |
|  | - | Siderophore-interacting protein |
| Phytohormone synthesis | *trp*C | Indole-3-glycerol phosphate synthase |
| Biocatalyst | *snp*A | Zinc-dependent metalloprotease |
|  | *fts*H, *htp*X | Zinc metalloprotease |
|  | *hya*D | Hydrogenase maturation protease |
|  | - | Lipase |
|  | *plc* | Phospholipase |
|  | *pnb*A | Carboxylesterase/lipase |
|  | - | Chitinase |
|  | *amy*A | Alpha-amylase |
|  | *kat*E | Catalase |
|  | *kat*G | Catalase/peroxidase |

2. Bioassays (In Progress)

Bioassays are currently being performed to confirm the multiple plant growth-promoting properties of *Streptomyces rochei* FE-3-1 indicated by genomic analysis. These assays assess phosphate solubilization, potassium solubilization, nitrogen assimilation, siderophore production, and indole-3-acetic acid (IAA) production.

3. Preparation of Spore Suspension (Completed)

Mature *Streptomyces* spores were selected using an inoculation loop and uniformly dispersed in sterile distilled water to prepare a spore suspension. The spore concentration was determined using a hemocytometer and diluted to the required application concentration.

4. Pot Experiment (Not Yet Done)

To evaluate the plant growth promotion activity of *Streptomyces rochei* FE-3-1 on rice, pot experiments will be conducted. Rice plants will be inoculated via root drenching with 30 mL of FE-3-1 spore suspension at different concentrations (10⁷, 10⁸, 10⁹ CFU/mL) per treatment. Control plants will receive 30 mL of sterile distilled water. Biometric properties (dry weight, fresh weight, root length, shoot length, and rice yield) will be measured and statistically analyzed. The experiment will include three independent trials. Each treatment will have five replicate pots, with each pot containing three rice plants.

Q-2: Lines 111-121: Please provide more details on the statistical methods used to validate the inhibition rates observed in the antagonistic activity assay against *Pyricularia oryzae*. Including specific statistical tests will add rigor to the data and help readers assess the reliability of the results. Additionally, have you assessed the ability of *Streptomyces rochei* FE-3-1 to control *Pyricularia oryzae*-caused disease in actual rice plants, beyond the in vitro antagonistic assays? If so, please provide detailed information on these trials, as it would greatly enhance the practical relevance of your findings.

R-2: Each treatment was replicated three times. Colony diameter was measured using the cross-measurement method with vernier calipers. Data are presented as means ± standard error, calculated using Excel 2010 (Microsoft Corporation, USA). All data were statistically analyzed using Duncan’s multiple range test (P = 0.05).
****(These additions appear on Page 6, Line 117-120 and Page 8, Lines 165–168; changes highlighted in red.)****

Statistical results demonstrated that the colony radius of *Pyricularia oryzae* on plates inoculated with antagonistic strain FE-3-1 was ****8.00 ± 0.035 mm****, whereas the control measured ****30.00 ± 0.046 mm****. This corresponds to an inhibition rate of ****73.33 ± 0.079%****.
****(These additions appear on Page 10, Lines 190–194; changes highlighted in red.)****

A limitation of this laboratory study is that it only confirms the antagonistic activity of *Streptomyces rochei* FE-3-1 against *Pyricularia oryzae* under controlled conditions. Antagonism observed in vitro may not necessarily extend to greenhouse or field environments. Therefore, greenhouse and growth chamber experiments are currently underway to further evaluate the efficacy of *Streptomyces rochei* FE-3-1 as a biocontrol agent. These trials will enhance the practical relevance of our findings.

Q-3:Lines 229-237: The identification of heavy metal resistance genes is intriguing, but it would be beneficial to discuss how these genes enhance the strain’s survival and effectiveness in agricultural environments that are subject to pollution or heavy metal contamination.

R-3: Soil heavy metal pollution is a significant limiting factor for bacterial survival in the environment. Bacterial mechanisms to counteract toxic metal contamination hold substantial environmental and economic importance. To ensure survival, bacteria have evolved diverse resistance and remediation strategies against heavy metals. Specific metabolic mechanisms immobilize heavy metals and reduce their bioavailability through sorption to cell components and exopolymers, transport and intracellular sequestration, or precipitation as insoluble organic and inorganic compounds. Additional processes include membrane filtration, ion exchange, adsorption, coagulation, oxidation-reduction, electrochemical treatment, evaporation, and chemical precipitation. These transformations—which alter heavy metal mobility and toxicity—carry immense biogeochemical significance and bioremediation potential.

The strain FE-3-1 harbors multiple putative functional proteins associated with heavy metals (cadmium, arsenic, copper) and zinc. These include transporters, P-type ATPases, resistance proteins, and others, conferring resistance to copper, zinc, cadmium, and arsenic. Such heavy metal resistance enhances FE-3-1’s ability to survive in extreme environments and provides a foundation for rhizosphere colonization and plant growth promotion.

[1] Pal A, Bhattacharjee S, Saha J, Sarkar M, Mandal P. Bacterial survival strategies and responses under heavy metal stress: a comprehensive overview. Crit Rev Microbiol. 2022;48(3):327-355. <https://doi.org/10.1080/1040841X.2021.1970512.> PMID: 34473592.

**The above contents have been added on Page 26-27 Line 467-479. The reference is cited on Page 40 Line 757-760, and the changes have been highlighted in red.**

Q-4: Lines 240-253: Could you clarify how the genes related to plant growth promotion, such as those for phosphorus and potassium solubilization, enhance the biocontrol efficiency of *Streptomyces rochei* FE-3-1? Understanding the synergy between plant growth and pathogen inhibition would be valuable.

R-4: According to Prokka annotations, the genome of *Streptomyces rochei* FE-3-1 harbors numerous genes associated with promoting plant growth, including those involved in phosphate solubilization, potassium solubilization, nitrogen assimilation and siderophore production.

Nitrogen, one of the most vital elements, plays an important role in plant growth and various metabolic activities. Conversion of nitrogen to ammonia (NH_3_) by *Streptomyces rochei* FE-3-1 using a nitrogenase protein complex. Phosphorus (P) is the second most vital macroelement after nitrogen and is required for adequate plant nutrition. It has key roles in the processes of photosynthesis, energy transfer, and various plant metabolic processes. In most soils, P is present in excess, but not in a form available to plants. only 0.1% of total soil phosphorus is available for plant use. Plant growth-promoting microbes offer a suitable option to enhance crop productivity. These microbes increase phosphorus availability in soil by mediating the mineralization of organic P in soil. Another vital macronutrient after nitrogen and phosphorus is potassium (K), which is the seventh most abundant element in the Earth’s crust. It plays a key role in various metabolic, growth, and development-related processes in plants. Moreover, potassium is responsible for activating more than 80 enzymes involved in photosynthesis activity, nitrate reduction, starch synthesis, and various energy metabolic processes. *Streptomyces rochei* FE-3-1 may function as biocontrol agents via a variety of mechanisms. An increase in plant vigor against various biotic stresses, diseases, and pests is also regulated by an adequate supply of N, P and K.

**The above contents have been added on Page 24 Line 429-433, and the changes have been highlighted in red.**

*Streptomyces rochei* FE-3-1 also functions as a biocontrol agent through multiple mechanisms. Siderophore synthesis—a crucial strategy for suppressing phytopathogens—involves iron-chelating compounds that sequester most iron in the rhizosphere. This iron limitation inhibits the proliferation of bacterial and fungal pathogens. Additionally, secondary metabolites produced by the bacterium may directly contribute to plant growth.

Q-5: Lines 257-274: The manuscript identifies several biosynthetic gene clusters, such as those for candicidin and streptothricin, which are related to antimicrobial activity. Expanding on how these clusters contribute to the biocontrol properties of *Streptomyces rochei* FE-3-1 would strengthen the link between the genomic data and the biological functions of the strain.

R-5: A total of 31 biosynthetic gene clusters were identified within the genome of *Streptomyces rochei* FE-3-1 via antiSMASH. Four of these biosynthetic gene clusters exhibited high similarity to known metabolites with antimicrobial properties such as candicidin, streptothricin, lipopeptide, and albaflavenone.

The candicidin complex was initially isolated from *Streptomyces griseus* IMRU3570. It demonstrates a potent inhibitory activity against fungi. The candicidin complex was regarded as the prototype of aromatic polyene macrolides. Similar to other polyketide polyenes, candicidin can interact with ergosterol existing in the membranes of fungi to form a transmembrane channel, resulting in K^+^ leakage and causing cell death.

Streptothricin is an N-glucoside antibiotic, which exists in different *Streptomyces* and is one of the earliest antibiotics isolated from *Streptomyces* and has broad-spectrum activity against Gram-positive, Gram-negative bacteria and pathogenic fungi. It inhibits protein biosynthesis in prokaryotic cells by inhibiting polypeptide synthesis via ribosome interactions.

Lipopeptide antibiotics represent the most recent addition to the antibiotic family, with molecular weights typically ranging from 1000-1600 Da. They are synthesized via a non-ribosomal multi-enzyme biosynthesis pathway. Surfactin, iturin, and fengycin are exemplary lipid peptides known for their intricate effects on plant pathogenic fungi.

The tricyclic sesquiterpene albaflavenone was first isolated from *Streptomyces albidoflavus*, and is known to exhibit antibacterial properties. The pathway for the biosynthesis of albaflavenone was recently elucidated in S. coelicolor A3(2).

Thus, the biosynthetic gene clusters provide a genetic foundation for FE-3-1’s biocontrol function, enabling it to deploy targeted antimicrobial warfare against agriculturally relevant pathogens, directly linking its genomic potential to its observed biological role in plant protection.

**The above contents have been added on Page 27-28 Line 493-506, and the changes have been highlighted in red.**

[1] Ji Z, Wang M, Zhang J, Wei S, Wu W. Two new members of streptothricin class antibiotics from Streptomyces qinlingensis sp. nov. J Antibiot (Tokyo). 2007; 60:739-744. <https://doi.org/10.1038/ja.2007.96.> PMID: 18276997

[2] Gil JA, Campelo-Diez AB. Candicidin biosynthesis in Streptomyces griseus. Appl. Microbiol. Biotechnol. 2003; 60: 633-642. <https://doi.org/10.1007/s00253-002-1163-9.> PMID: 12664141

[3] Zhao B, Lin X, Lei L, Lamb DC, Kelly SL, Waterman MR, et al. Biosynthesis of the sesquiterpene antibiotic albaflavenone in Streptomyces coelicolor A3(2). J. Biol. Chem. 2008; 283: 8183-8189. <https://doi.org/10.1074/jbc.M710421200.> PMID: 18234666

[4] Stein T. Bacillus subtilis antibiotics: structures, syntheses and specific functions. Mol. Microbiol. 2005; 56(4):845-857. <https://doi.org/10.1111/j.1365-2958.2005.04587.x.> PMID: 15853875

[5] Kim PI, Ryu J, Kim YH, Chi YT. Production of biosurfactant lipopeptides Iturin A, fengycin and surfactin A from Bacillus subtilis CMB32 for control of Colletotrichum gloeosporioides. J Microbiol Biotechnol. 2010; 20(1):138-145. <https://doi.org/10.4014/jmb.0905.05007.> PMID: 20134245

[6] Lam VB, Meyer T, Arias AA, Ongena M, Oni FE, Höfte M. Bacillus cyclic lipopeptides Iturin and Fengycin control rice blast caused by Pyricularia oryzae in potting and acid sulfate soils by direct antagonism and induced systemic resistance. Microorganisms. 2021; 9(7):1441. <https://doi.org/10.3390/microorganisms9071441.> PMID: 34361878

[7] Liao JH, Chen PY, Yang YL, Kan SC, Hsieh FC, Liu YC. Clarification of the antagonistic effect of the lipopeptides produced by Bacillus amyloliquefaciens BPD1 against Pyricularia oryzae via in situ MALDI-TOF IMS analysis. Molecules. 2016; 21(12):1670. <https://doi.org/10.3390/molecules21121670.> PMID: 27918491

[8] Omoboye OO, Geudens N, Duban M, Chevalier M, Flahaut C, Martins JC, et al. Pseudomonas sp. COW3 produces new bananamide-type cyclic lipopeptides with antimicrobial activity against Pythium myriotylum and Pyricularia oryzae. Molecules. 2019; 24(22):4170. <https://doi.org/10.3390/molecules24224170.> PMID: 31744250

Q-6: Lines 257-277: It would be helpful to include a comparison of the identified secondary metabolites with known commercial biocontrol agents in terms of their effectiveness and range of activity. This could give a clearer perspective on the potential competitive advantage of *Streptomyces rochei* FE-3-1.

R-6: Based on different *Streptomyces* strains, many commercial biocontrol agents, such as Actinovate (*Streptomyces lydicus* WYEC108), Rhizovit (*Streptomyces rimosus*), and Mycostop (*Streptomyces griseoviridis* K61), have been developed for agricultural purposes, and these fungicides show resistance to a variety of pathogens (S6 Table). Therefore, *Streptomyces* are important resources for agricultural fungicides or biofertilizers. In this study, a total of 31 biosynthetic gene clusters were identified within the genome of *Streptomyces rochei* FE-3-1, its metabolites exhibited potent antagonistic activity against plant pathogen *Pyricularia oryzae* by the agar disk diffusion method. Four of these biosynthetic gene clusters exhibited high similarity to known metabolites with antifungal and antibacterial properties such as candicidin, streptothricin, lipopeptide, and albaflavenone. Some gene clusters were also related to active metabolites such as isorenieratene, desferrioxamine, hopene, ectoine, sapB, fluostatins, 7-prenylisatin, melanin, borrelidin and geosmin. Therefore, this strain is predicted to have inhibitory activities against a variety of pathogens, and we will further explore these active metabolites in the future.

S6 Table. Commercialized *streptomyces* based biopesticides

| Biocontrol agent(s) | Active ingredient | Target pathogen(s) | Biocontrol  mechanism | Country |
| --- | --- | --- | --- | --- |
| Actinovate | s*treptomyces lydicus* WYEC108 | *Fusarium* spp., *Rhizoctonia* spp.,  *Pythium* spp., *Phytophthora*  spp., *Erisiphe* spp., *Sphaeroteca*  spp., *Laveillula* spp., *Sclerotinia*  spp. | Antibiosis and  hyperparasitism | European Union |
| Rhizovit | *Streptomyces rimosus* | *Pythium* spp., *Fusarium* spp.,  *Phomopsis* spp., *Phytophthora*  spp., *R. solani*, *A. brassicola*,  *Botrytis* spp., *Fusarium* spp. | Antibiosis | - |
| Mycostop | *Streptomyces griseoviridis* K61 | *Ceratocystis radicicola*,  *Alternaria* spp., *Rhizoctonia*  *solani*, *Fusarium* spp.,  *Phytophthora* spp., *Pythium* spp. | Competition,  hyperparasitism, and  antibiosis | Canada |

[1] Marine SC, Newark MJ, Korir RC, Everts KL. Evaluation of Rotational Biopesticide Programs for Disease Management in Organic Cucurbit Production. Plant Dis. 2016;100(11):2226-2233. <https://doi.org/10.1094/PDIS-02-16-0252-RE.> PMID: 30682910

[2] Himmelstein JC, Maul JE, Everts KL. Impact of Five Cover Crop Green Manures and Actinovate on *Fusarium Wilt* of Watermelon. Plant Dis. 2014 Jul;98(7):965-972. <https://doi.org/10.1094/PDIS-06-13-0585-RE.> PMID: 30708841

[3] Minuto A, Spadaro D, Garibaldi A, Gullino M L. Control of soilborne pathogens of tomato using a commercial formulation of *Streptomyces griseoviridis* and solarization. Crop Protection, 2006, 25: 468-475. https://doi.org/10.1016/j.cropro.2005.08.001

[4] Silva GC, Kitano IT, Ribeiro IAF and Lacava PT. The Potential Use of

*Actinomycetes* as Microbial Inoculants and Biopesticides in Agriculture. Front. Soil Sci. 2022; 2:833181. https://doi.org/10.3389/fsoil.2022.833181.

**The above contents have been added on Page 28-29 Line 516-525, The reference is cited on Page 36 Line 673-676, and S6 Table has been inserted into the supplementary materials on Page 31 Line 560, and the changes have been highlighted in red.**

Q-7: Lines 333-362: The manuscript mentions unique homologous genes found in *Streptomyces rochei* FE-3-1. It would be beneficial to discuss how these genes might contribute to the strain’s unique abilities or adaptations, especially in comparison with other *Streptomyces* species.

R-7: In this study, comparative genomic analysis of nine distinct *Streptomyces* strains demonstrated that 244 unique gene clusters were specific to the strain FE-3-1. Among these unique homologous genes, such as nitrite reductase [EC:1.7.2.1], pyruvate carboxylase [EC:6.4.1.1], and polyphosphate kinase [EC:2.7.4.34], etc. The enzyme proteins encoded by these gene clusters play crucial roles in promoting growth, synthesizing secondary metabolites and tolerating external environmental stress. Moreover, these unique homologous genes may contribute to the strain's distinctive capabilities.

These unique homologous genes are not merely genetic signatures; they encode functional machinery directly contributing to FE-3-1's core physiology and ecological strategy. The nitrite reductase expands nitrogen metabolic options, the pyruvate carboxylase fortifies central carbon metabolism for growth and biosynthesis, and the polyphosphate kinase bolsters stress resistance and metabolic regulation. Together, they provide a compelling genetic basis for the observed uniqueness of *S. rochei* FE-3-1 compared to other *Streptomyces* species, likely enabling its survival, adaptation, and specialized functions within complex microbial communities. Further functional characterization of these and other unique clusters will precisely define their roles in the strain's distinctive capabilities.

**The above contents have been added on Page 23 Line 402-410, and the changes have been highlighted in red.**

Q-8: Lines 364-374: Finally, your study contributes significantly to microbial genome mining. Could you emphasize how your findings advance the field of genome-guided discovery of biocontrol agents and antibiotics?

R-8: The advent of the genomic era has unlocked immense potential for discovering novel natural products. Microbial genome mining offers a powerful alternative to traditional, low-throughput discovery methods, enabling the identification of secondary metabolites that serve as vital scaffolds for combinatorial biosynthesis. These engineered compounds hold significant promise for crop protection and diverse biotechnological applications.

Traditional methods often fail to uncover many microbial metabolites because the associated biosynthetic genes remain cryptic, frequently requiring specific induction conditions for expression. Genome mining directly addresses this limitation by facilitating the systematic identification and characterization of these cryptic, unannotated biosynthetic gene clusters (BGCs), thereby revealing pathways likely to produce novel compounds.

Among microorganisms, bacteria—owing to their relatively small and simple genomes—have been the primary focus of genome mining efforts. Notably, *Streptomyces* spp., already renowned for prolific secondary metabolite production, have been found through genome sequencing to encode an even greater wealth of potential metabolites. This potential is underscored by the staggering abundance of Actinomycetes in the environment: an estimated ~10^26^ colony-forming units exist in just the top 10 cm of global soil. Yet, over the past fifty years, only a minute fraction (~10^7^) have been screened for metabolite production. Whole-genome sequencing and advanced bioinformatics tools are now unveiling vast numbers of previously unknown metabolites from diverse bacterial sources.

This study provides comprehensive insights into the genomic architecture and functional potential of *Streptomyces rochei* strain FE-3-1. The FE-3-1 genome harbors a rich repertoire of diverse BGCs, indicative of a substantial capacity to biosynthesize novel natural products. These findings highlight the exceptional potential of this strain to produce a wide array of metabolites, potentially including novel antibiotics or antimicrobial agents effective against agricultural fungal pathogens. Consequently, *S. rochei* FE-3-1 emerges as an outstanding candidate for mining novel natural products. Ultimately, decoding microbial genomes and harnessing their encoded natural products offers a transformative approach to addressing critical challenges in agriculture.

[1] Kenshole E, Herisse M, Michael M, Pidot SJ. Natural product discovery through microbial genome mining. Curr Opin Chem Biol. 2021;60:47-54. https://doi.org/10.1016/j.cbpa.2020.07.010. PMID: 32853968

[1] Bachmann BO, Van Lanen SG, Baltz RH. Microbial genome mining for accelerated natural products discovery: is a renaissance in the making? J Ind Microbiol Biotechnol. 2014;41(2):175-84. <https://doi.org/10.1007/s10295-013-1389-9.> PMID: 24342967
[2] Tamang P, Upadhaya A, Paudel P, Meepagala K, Cantrell CL. Mining Biosynthetic Gene Clusters of *Pseudomonas vancouverensis* Utilizing Whole Genome Sequencing. Microorganisms. 2024; 12(3): 548. <https://doi.org/10.3390/microorganisms12030548.> PMID: 38543599.

**The above contents have been added on Page 22 Line 376-382, the reference is cited on Page 37 Line 696-699, and the changes have been highlighted in red.**

Q-9: Introducing biocontrol agents into agricultural systems carries some risk. Have you considered any potential environmental impacts or risks associated with using *Streptomyces rochei* FE-3-1? If so, how might these be mitigated?

R-9: The introduction of any novel biological control agent, including *Streptomyces rochei* FE-3-1 (FE-3-1), necessitates rigorous evaluation of potential environmental risks alongside its benefits.

(1) **Non-Target Effects on Soil Microbiota**

**Risk:** Soil ecosystems harbor immense microbial diversity crucial for nutrient cycling, soil structure, and plant health. FE-3-1, being a prolific producer of antimicrobial compounds, could inhibit or eliminate beneficial non-target bacteria, fungi, actinomycetes, or protozoa. This disruption could alter microbial community composition and function, potentially reducing soil fertility, suppressing natural disease suppression mechanisms, or favoring unintended opportunistic pathogens.

**Mitigation:** Deep characterization of FE-3-1's secondary metabolite profile under field-relevant conditions is essential. Identifying the specific compounds produced and their ecological concentrations allows for targeted risk assessment.

(2) **Horizontal Gene Transfer (HGT)**

**Risk:** *Streptomyces* are known for their genetic plasticity and ability to exchange DNA via HGT (conjugation, transduction). There is a risk that genes encoding antimicrobial resistance, virulence factors, or novel biosynthetic pathways could be transferred from FE-3-1 to indigenous soil bacteria, including potential human or animal pathogens. This is particularly concerning if FE-3-1 produces clinically relevant antibiotics.

**Mitigation:**Thorough genome sequencing and annotation of FE-3-1 to identify any mobile genetic elements (plasmids, transposons, integrative conjugative elements) and genes associated with antimicrobial resistance, pathogenicity, or HGT machinery.

(3) **Disruption of Existing Ecological Balances**

**Risk:** Introducing a potent antimicrobial producer could shift competitive balances in the soil microbiome in unpredictable ways. Suppressing one pathogen might inadvertently favor the emergence of a different, potentially worse, pathogen that was previously kept in check by the suppressed organism or microbial community.

**Mitigation:** Implementing careful, phased introduction starting with small-scale field trials to observe ecological effects before large-scale deployment.

In this study, Streptomyces rochei strain FE-3-1 was isolated from rice rhizosphere soil. As an indigenous rhizosphere microorganism of rice, its reintroduction into the rhizosphere following propagation poses low environmental risk. Laboratory safety assessments, including hemolysis testing on blood plates, yielded negative results (Figure 1), further supporting the strain's low environmental risk potential. To ensure comprehensive environmental safety during subsequent product development, rigorous animal testing should be implemented.

Figure 1 Hemolysis experiment of the strain FE-3-1

1, 2, 3: The strain FE-3-1
