# Supplementary material for: Isolation, identification, and whole-genome sequencing of Streptomyces rochei FE-3-1 against Pyricularia oryzae
Source: PLoS One. 2025 Oct 29;20(10):e0331386. doi: 10.1371/journal.pone.0331386 (PMC12571248; doi:10.1371/journal.pone.0331386)
Supplement: Attachment — Submitted filename: Response_to_Reviewers_auresp_4.doc [file pone.0331386.s006.doc]

Dear editors,

Thank you very much for your E-mail with regard to our manuscript (PONE-D-24-32365R3) together with the comments from the reviewers. The comments from the reviewers were very helpful. We have responded each comment from the reviewers in a separate letter and taken all of these comments into account in preparing the revised manuscript. All of updates in the manuscript were marked via changing the font colour of the text. We believe that manuscript has been revised satisfactorily and hope it will be accepted for publication in PLOS ONE.

We thank again the reviewers for the helpful comments. Should you require any further information, please do not hesitate to ask.

Thanks very much for your attention and consideration.

Sincerely yours

Shiping Shan

**Responses to reviewers’ comments**

(Q refers to the question and R for the reply)

**To the Comments of Reviewer**

Q-1: The supplementary table (S6) provides an overview of commercial benchmarks. It might be helpful to briefly discuss in the main text how the observed inhibition rates and genomic potential of FE-3-1 compare quantitatively, where possible, to those products. Also it is unclear which of references 1-4 relates to respective preparation.

R-1: The following contents have been added on Page 30-31 Line 539-554, and the changes have been highlighted in red.

While this study demonstrates the potent *in vitro* antagonism of *Streptomyces rochei* FE-3-1 metabolites against *Pyricularia oryzae*, a direct quantitative comparison of inhibition rates to commercial products like Actinovate (*Streptomyces lydicus*), Rhizovit (*Streptomyces* sp.), or Mycostop (*Streptomyces griseoviridis*) requires further bioassay against a common pathogen panel. However, the genomic analysis reveals FE-3-1 possesses significant potential comparable to these commercially exploited strains. Its genome harbors 31 biosynthetic gene clusters, at least four linked to known potent antimicrobials. This genomic richness suggests FE-3-1 has the inherent capacity to produce a diverse arsenal of bioactive compounds, potentially explaining its observed activity and supporting its potential to resist a wide range of plant pathogens. This preliminary genomic evidence positions FE-3-1 as a strong candidate for development into novel biofertilizers or biopesticides, warranting further exploration of its metabolite production.

References 1 to 4 have been respectively cited at appropriate positions in the main text.

The reference 1 has been cited on Page 3 Line 45, and the changes have been highlighted in red.

The reference 2 has been cited on Page 3 Line 48, and the changes have been highlighted in red.

The reference 3 has been cited on Page 3 Line 51, and the changes have been highlighted in red.

The reference 4 has been cited on Page 3 Line 53, and the changes have been highlighted in red.

The reference 5 has been cited on Page 3 Line 56, and the changes have been highlighted in red.

Q-2: English: there are minor issues with grammar and phrasing throughout the text. A careful editing may be necessary. Although my judgment may be subjective due to the fact that I’m not a native speaker.

R-2: All of us have revised the whole manuscript and corrected the grammatical and phrasing errors.

The word "has" has been changed to "have" on Page 3 Line 47, and the changes have been highlighted in red.

The word "bacteria" has been changed to "bacterial species" on Page 3 Line 58, and the changes have been highlighted in red.

The sentence "They also play a crucial role as growth promotion and biocontrol agents, and their use in agriculture is becoming increasingly widespread" has been changed to "They also play a crucial role as agents for growth promotion and biocontrol, with their application in agriculture becoming increasingly prevalent" on Page 3 Line 58-60, and the changes have been highlighted in red.

The phrase "Prokka annotated" has been changed to "Prokka- annotated" on Page 8 Line 149-150, and the changes have been highlighted in red.

The word "identify" has been changed to " identified" on Page 19 Line 309, and the changes have been highlighted in red.

The word "with" has been changed to " and" on Page 20 Line 328, and the changes have been highlighted in red.

The phrase "S6 Table" has been changed to "Table 6" on Page 29 Line 520, and the changes have been highlighted in red.

Q-3: If possible, reference supporting data (statistical outputs, graphical results) directly in the main text rather than relying on supplementary figures..

R-3: The supporting reference data S6 has been relocated to the main text and is now designated as Table 6.

Table 6 Commercialized *streptomyces* based biopesticides^[19]^

| **Biocontrol agent(s)** | **Active ingredient** | **Target pathogen(s)** | **Biocontrol**  **mechanism** | **Country** |
| --- | --- | --- | --- | --- |
| **Actinovate** | s*treptomyces lydicus* WYEC108 | *Fusarium* spp., *Rhizoctonia* spp.,  *Pythium* spp., *Phytophthora*  spp., *Erisiphe* spp., *Sphaeroteca*  spp., *Laveillula* spp., *Sclerotinia*  spp. | Antibiosis and  hyperparasitism | European Union |
| **Rhizovit** | *Streptomyces rimosus* | *Pythium* spp., *Fusarium* spp.,  *Phomopsis* spp., *Phytophthora*  spp., *R. solani*, *A. brassicola*,  *Botrytis* spp., *Fusarium* spp. | Antibiosis | - |
| **Mycostop** | *Streptomyces griseoviridis* K61 | *Ceratocystis radicicola*,  *Alternaria* spp., *Rhizoctonia*  *solani*, *Fusarium* spp.,  *Phytophthora* spp., *Pythium* spp. | Competition,  hyperparasitism, and  antibiosis | Canada |

The above table has been added on Page 30 Line 534-537, and the changes have been highlighted in red.

Q-4: In the discussion, clearly differentiate between what is demonstrated (in vitro/in silico) and what is still hypothetical or future work (in planta, field results, compound isolation, commercial development).

R-4: Our study demonstrates *in vitro* that *Streptomyces rochei* FE-3-1 inhibits the plant pathogen *Pyricularia oryzae* on agar plates, confirming its biocontrol potential. *In silico* genomic analysis revealed 31 biosynthetic gene clusters (BGCs), including 5 PKS/PKS-like clusters, and identified genes encoding hydrolytic enzymes (proteases, lipases, amylase, chitinase) potentially involved in fungal cell wall degradation. Comparative genomics showed 244 unique gene clusters in FE-3-1, including those encoding nitrite reductase, pyruvate carboxylase, and polyphosphate kinase, which may contribute to environmental stress tolerance and secondary metabolism. KEGG pathway analysis *in silico* further identified genes linked to plant-beneficial functions: nitrogen assimilation (nitrate reduction to ammonia), phosphate/potassium solubilization, and sulfur metabolism (hydrogen sulfide production via enzymes CysN, CysD, CysC, CysH, SirA). Three siderophore BGCs (clusters 12, 19, 22) were annotated, with cluster 12 showing 83% similarity to desferrioxamine B/E clusters. Heavy metal resistance genes were also predicted *in silico*, suggesting environmental adaptability.

In contrast, the functional and ecological roles of these genetic elements remain largely hypothetical or require future validation:

Plant growth promotion mechanisms (nitrogen fixation, P/K solubilization, H₂S signaling, siderophore-mediated iron uptake) and biocontrol efficacy are predicted *in silico* but not yet verified in planta or in field settings.

Metabolite activity: While BGCs for streptothricin, candicidin, albaflavenone, and lipopeptides were identified *in silico*, these compounds have not been isolated from FE-3-1, and their bioactivity is inferred from literature.

Biocontrol applicability:The strain’s proposed broad-spectrum pathogen inhibition and commercial potential (analogous to Actinovate® or Mycostop®) are hypotheses requiring in planta trials, formulation studies, and field efficacy data.

Environmental remediation: Heavy metal immobilization mechanisms are proposed based on genomic annotation but lack experimental confirmation.
 Thus, future work must prioritize compound isolation, biochemical characterization, in planta efficacy testing, and field validation to translate genomic potential into practical applications.

Q-5: Reinforce the conclusion with perspective on how this genome resource could contribute to the mining of new actinobacterial biocontrol strains.

R-5: The conclusion section was revised and reorganized to emphasize the potential of this genomic resource in facilitating the discovery of novel actinomycetes-based biological control strains.

The following contents have been added on Page 30-31 Line 539-554, and the changes have been highlighted in red.

This study isolated a *Streptomyces* strain, FE-3-1, from the rice rhizosphere, demonstrating significant inhibitory activity against the plant pathogen *Pyricularia oryzae*. Morphological and phylogenetic analysis identified the strain as *Streptomyces rochei*. Whole genome sequencing revealed a substantial biosynthetic potential, anchored by 31 biosynthetic gene clusters including at least four associated with known antimicrobials. This high-quality genome sequence provides a crucial resource for comparative actinobacterial genomics. Genome mining confirmed the strain's strong capacity for secondary metabolite production, highlighting its value as a source of bioactive compounds. Collectively, these findings provide a robust theoretical foundation for developing *Streptomyces rochei* FE-3-1 into effective biofertilizers or biopesticides. Furthermore, the genomic insights facilitate the targeted mining of related, underexplored actinobacteria, paving the way for discovering next-generation biocontrol strains with novel or enhanced modes of action against diverse plant pathogens.
